# Bioassay-Guided Fractionation of *Siparuna glycycarpa* *n*-Butanol Extract with Inhibitory Activity against Influenza A(H1N1)pdm09 Virus by Centrifugal Partition Chromatography (CPC)

**DOI:** 10.3390/molecules27020399

**Published:** 2022-01-08

**Authors:** Carla Monteiro Leal, Suzana Guimarães Leitão, Leonardo Luiz Oliveira de Mello, Isabel de Castro Rangel, Carlos Vinicius Azevedo da Silva, Milene Dias Miranda, Amanda Resende Tucci, Camilla Blanco de Assis, Carolina de Queiroz Sacramento, Natalia Fintelman-Rodrigues, Hector Henrique Ferreira Koolen, Boniek Gontijo Vaz, Rosineide Costa Simas, Gilda Guimarães Leitão

**Affiliations:** 1 Programa de Pós-Graduação em Biotecnologia Vegetal e Bioprocessos (PBV), Centro de Ciências da Saúde, Universidade Federal do Rio de Janeiro, Rio de Janeiro 21941-902, Brazil; carlam.leal@yahoo.com.br; 2 Instituto de Pesquisas de Produtos Naturais, Centro de Ciências da Saúde, Universidade Federal do Rio de Janeiro, Rio de Janeiro 21941-902, Brazil; leonardo_luiz_@outlook.com (L.L.O.d.M.); isabeldecastror@gmail.com (I.d.C.R.); 3 Faculdade de Farmácia, Centro de Ciências da Saúde, Universidade Federal do Rio de Janeiro, Rio de Janeiro 21941-902, Brazil; sgleitao@pharma.ufrj.br; 4 Grupo de Pesquisas em Metabolômica e Espectrometria de Massas, Escola Superior de Ciências da Saúde, Universidade do Estado do Amazonas, Manaus 69065-000, Brazil; cv25066@gmail.com (C.V.A.d.S.); hkoolen@uea.edu (H.H.F.K.); 5 Laboratório de Vírus Respiratórios e do Sarampo, Instituto Oswaldo Cruz, Fundação Oswaldo Cruz, Rio de Janeiro 21041-210, Brazil; milenediasmiranda@gmail.com (M.D.M.); biologa.t@gmail.com (A.R.T.); 6 Laboratório de Imunofarmacologia, Instituto Oswaldo Cruz, Fundação Oswaldo Cruz, Rio de Janeiro 21041-210, Brazil; camillablanco.a@gmail.com (C.B.d.A.); carol.qsacramento@gmail.com (C.d.Q.S.); nataliafintelman@gmail.com (N.F.-R.); 7 Centro de Desenvolvimento Tecnológico em Saúde, Instituto Nacional de Ciência e Tecnologia de Gestão da Inovação em Doenças Negligenciadas, Fundação Oswaldo Cruz, Rio de Janeiro 21041-210, Brazil; 8 Laboratório de Cromatografia e Espectrometria de Massas (LaCEM), Instituto de Química, Universidade Federal de Goiás, Goiânia 74690-900, Brazil; boniek@gmail.com (B.G.V.); simas.rc@gmail.com (R.C.S.)

**Keywords:** centrifugal partition chromatography, influenza virus, mass spectrometry, off-line LC-MS/MS, Siparunaceae, solvent system

## Abstract

*Siparuna glycycarpa* occurs in the Amazon region, and some species of this genus are used in Brazilian folk medicine. A recent study showed the inhibitory effect of this species against influenza A(H1N1)pdm09 virus, and in order to acquire active fractions, a polar solvent system *n*-butanol-methanol-water (9:1:10, *v*/*v*) was selected and used for bioassay-guided fractionation of *n*-butanol extract by centrifugal partition chromatography (CPC). The upper phase was used as stationary phase and the lower phase as mobile (descending mode). Among the collected fractions, the ones coded SGA, SGC, SGD, and SGO showed the highest antiviral inhibition levels (above 74%) at 100 µg·mL^−1^ after 24 h of infection. The bioactive fractions chemical profiles were investigated by LC-HRMS/MS data in positive and negative ionization modes exploring the Global Natural Products Social Molecular Networking (GNPS) platform to build a molecular network. Benzylisoquinoline alkaloids were annotated in the fractions coded SGA, SGC, and SGD collected during elution step. Aporphine alkaloids, *O*-glycosylated flavonoids, and dihydrochalcones in SGO were acquired with the change of mobile phase from lower aqueous to upper organic. Benzylisoquinolinic and aporphine alkaloids as well as glycosylated flavonoids were annotated in the most bioactive fractions suggesting this group of compounds as responsible for antiviral activity.

## 1. Introduction

Influenza viruses are one of the most relevant etiological agents of acute respiratory infections. Influenza A viruses are highly mutagenic and can infect both animals and humans, causing seasonal epidemics and pandemics, for instance the influenza A(H1N1) 2009 pandemic, which led to significant morbidity, mortality rates, and burden to public health [1,2]. In July 2020, during the worldwide pandemic of COVID-19, an outbreak of another influenza A subtype, A(H3N2), occurred in the Kingdom of Cambodia. Among the Pagoda residents, 82.5% tested positive for this virus [3]. Therefore, the search for anti-influenza compounds that may prevent and treat serious infections and reduce transmissibility is pivotal.

Plants and their derived natural products could be a source for new antiviral drugs [4,5]. Some species of the genus *Siparuna* are used in Brazilian folk medicine [6,7], and recently, our group showed that the extracts from Amazonian *Siparuna* species presented inhibitory effect against influenza A(H1N1)pdm09 virus replication [8]. The *n*-butanol extracts from *Siparuna glycycarpa* (SG) and *Siparuna sarmentosa* (SS) demonstrated high antiviral activity, inhibiting, respectively, 96.0 ± 1.3% and 89.5 ± 0.8% of influenza virus replication 24 h post-infection. The selective index (SI) values for SG and SS were 40 and >27, higher than those for other *n*-butanol extracts published in the literature [8,9]. Alkaloids, *O*- and *C*-flavonoid glycosides, dihydrochalcones, and a procyanidin dimer were annotated in these extracts, and according to the literature, they have been demonstrated as potential inhibitors of influenza virus [8,10,11].

Plant extracts are generally complex mixtures composed of many different compounds within the several classes of secondary metabolites. According to ethnopharmacological knowledge, the bio-guided fractionation approach has been employed for the screening and fractionation of extracts in natural products research for the obtention of bioactive fractions and identification of active compounds [12,13]. Many chromatographic techniques can be employed in this approach. However, depending on the applied technique, some bioactive compounds may be lost due to irreversible adsorption on the chromatographic column, common in solid stationary phase techniques [14,15,16,17].

Thus, a liquid–liquid extraction method such as countercurrent chromatography (CCC) is an excellent alternative, as it is a liquid–liquid partition chromatography technique in which the stationary liquid phase is retained in a column without the use of solid supports. CCC is extremely useful in the purification of compounds from complex matrices such as plant extracts. In modern CCC, the equipment operates in a centrifugal way. The rotational movement of the column can be of two types, generating hydrostatic equilibrium between the two liquid phases or the hydrodynamic equilibrium. Centrifugal partition chromatography (CPC) uses a hydrostatic column with a constant-gravity force field produced by a single-axis rotating column [18,19]. The sample properties, solvent system choice, instrument, and instrumental method influence the efficiency within the separation procedure by CPC [20]. Among the advantages of the hydrostatic techniques such as high-flow rates working possibility, one stands out, which is the excellent retention of all solvent systems, especially of those composed of high-polarity and viscous solvents, including the aqueous two-phase solvent systems (ATPS), which are not retained in hydrodynamic columns [18,20].

CPC has been applied in different natural products studies for the isolation and purification of compounds, and for obtaining simplified fractions from crude extracts [19,21,22,23]. The off-line coupling of CPC with liquid chromatography–mass spectrometry is an efficient tool for giving access to the chemical profile of fractions [24]. By employing tandem mass spectrometry, it is possible to annotate the known and unknown compounds through spectra fragmentation data [14,24].

In this study, the *n*-butanol extract from *S. glycycarpa*, previously shown to be active against influenza A(H1N1)pdm09 replication, was fractionated by CPC using *n*-butanol-methanol-water (BuMWat), a polar solvent system that failed stationary phase retention on hydrodynamic equipment. The acquired fractions were evaluated for their inhibitory effects against influenza A(H1N1) virus replication, and the bioactive fractions were analyzed by LC-HRMS/MS and molecular networking for the compound’s annotation.

## 2. Results and Discussion

### 2.1. Centrifugal Partition Chromatography of the n-Butanol Extract from S. glycycarpa

The choice of an adequate solvent system is essential for the success of fractionation in CCC. When dealing with polar extracts, the composition of a suitable solvent system generally involves a ternary mixture of polar organic solvents such as ethyl acetate, *n*-butanol, a short-chain alcohol, and water [24,25]. The *n*-butanol–methanol–water (BuMWat) solvent system is a hydrophilic-organic solvent system that can be applied for the purification of some peptides, glycans, phenolic acids, glycosylated polyphenols, and hydrophilic alkaloids [20,23,26,27]. The presence of high-viscosity solvents such as *n*-butanol, results in lower retention (or even no retention at all) of the stationary phase in equipment with a hydrodynamic column as in high-speed countercurrent chromatography (HSCCC), affecting chromatographic peak resolution [23]. Thus, better stationary phase control is one of the advantages of CPC over HSCCC, which allow using hydrophilic systems [20].

Previous chemical profiling of the extract under study by ion-trap liquid chromatography–high resolution mass spectrometry revealed its complex composition, containing different compounds of high polarity [8]. Thus, a solvent system such as *n*-butanol–methanol–water (BuMWat) would be suitable for covering the large range of high polarity of the compounds present in this extract and was proposed for its bioguided fractionation. To do this, different volume ratios of *n*-butanol–methanol–water were tested: (1) 10:0:10, *v/v*, (2) 9:1:10, *v/v*, and (3) 8:2:10, *v/v* (Supplementary Figure S1). Solvent system 2 was selected, as it provided good solubility of the extract and homogenous distribution of compounds between the two liquid phases. Due to compounds of interest being mainly found in the upper organic phase, this phase was chosen as stationary and the lower aqueous phase as mobile (descending mode).

Three 500 mg fractionations of the *n*-butanol extract from *S. glycycarpa* were performed in the elution–extrusion mode, allowing the recovery of retained compounds in the stationary phase [28]. During the first experiment 47 fractions of 12 mL were collected with a flow rate of 6 mL·min^−1^ and a rotor spinning at 1000 rpm (Supplementary Figure S2). In these conditions a loss of stationary phase (102 mL) was observed in the first collected tubes (1–17) and, consequently, a low retention of stationary phase (59.2%). Flow rate and rotation speed are essential for the fluid dynamics in CPC and can influence the quality of separation [24,29]. Therefore, a second fractionation was carried out with the same conditions, adjusting the rotation to 1300 rpm to improve stationary phase retention. Indeed, there was an increase in the stationary phase retention to 70% and an improvement in the chromatographic separation (Supplementary Figure S3). Through the CPC–UV chromatogram of collected fractions it was possible to monitor (UV detection) the presence of compounds with absorption bands at about 220 nm and 280 nm in the elution step (fractions 7 to 12), which may account for alkaloids [30]. Fractions 33 to 40, collected during the extrusion step, showed the presence of absorption maxima that ranged from 300–380 nm and 240–285 nm, characteristic, respectively, of bands I and II of flavonoids [31] (Supplementary Figure S4).

Aiming at improving the chromatographic separation, the local minimum function was applied in the last fractionation. This tool of the Glider CPC software moves the probe to the next tube during a collection phase when an inflection of the signal appears on the chromatogram, allowing us to separate fractions in different tubes regardless of their volume and, although an accurate prediction of sample volume and tube quantity, as in a conventional CPC separation does not seem to be observed, it can help to separate poorly resolved chromatographic peaks.

For this, the rotor spinning was adjusted to 1300 rpm with a flow rate of 6 mL·min^−1^. The stationary phase retention was maintained at 70%, and 167 fractions were obtained with the local minimum function. Although a larger number of fractions were collected, TLC analysis showed an improvement in the separation profile (Supplementary Figure S5). The obtained fractions with different chemical compounds distribution were pooled into 18 final fractions, according to their chemical and chromatographic similarities by TLC profile (Supplementary Table S1). The anti-influenza activity of the simplified fractions was evaluated to target the active fractions and the chemical compounds annotation through LC-HRMS/MS analysis.

### 2.2. In Vitro Anti-Influenza Activity of Fractions Acquired by CPC

The 18 final fractions from the third fractionation of the *n*-butanol extract from *S. glycycarpa* were first screened for their cytotoxicity at 100 µg·mL^−1^, evaluated in Madin-Darby Canine Kidney (MDCK) cells by the MTT (3-[4,5-dimethylthiazol-2-yl]-2,5 diphenyl tetrazolium bromide) assay. Most of the fractions, as well as the crude *n*-butanol extract (SGBu) and OST-car (control), were not cytotoxic and maintained cell viability above 80%, except for SGJ, SGL, SGM, SGN, SGP, SGQ, and SGR, which reduced cell viability to values below 50% (Supplementary Table S2).

**Figure 1:**
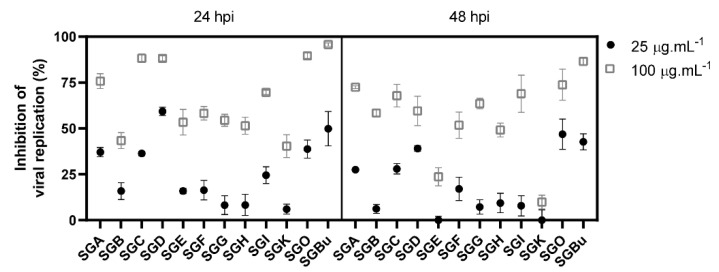
Inhibition of influenza virus replication by CPC pooled fractions with cell viability above 80%.

In the sequence, viral replication inhibition assays were performed with the least cytotoxic fractions (cell viability > 80%) to verify their antiviral activity against influenza virus. MDCK cells were infected with influenza A(H1N1) virus and then treated for 24 and 48 hours post-infection (hpi) with concentrations of 25 and 100 µg·mL^−1^ of the fractions, which, based on our previous results of *S. glycycarpa n*-butanol extract (SGBu) EC_50_ [8], were expected to inhibit virus replication by 50 and 90%, respectively. The results showed that the treatment with 100 µg·mL^−1^ of the fractions, for 24 h, was more potent than that with 25 µg·mL^−1^ (Figure 1 and Supplementary Table S3). The fractions SGA, SGC, SGD, and SGO presented the highest inhibition levels (above 74%) at a concentration of 100 µg·mL^−1^. Although less pronounced, viral replication inhibition was maintained until 48 h of treatment with 100 µg·mL^−1^ (Figure 1 and Supplementary Table S3). The clinically approved anti-influenza drug oseltamivir (OST) and the previously described SGBu were used as positive controls and inhibited influenza replication by 100 and 95% at 100 µg·mL^−1^, respectively, after 24 hpi, remaining with high percentages of inhibition even after 48 hpi (Figure 1 and Supplementary Table S3).

### 2.3. LC-HRMS/MS and Molecular Networking Analyses of CPC Fractions

**Figure 2:**
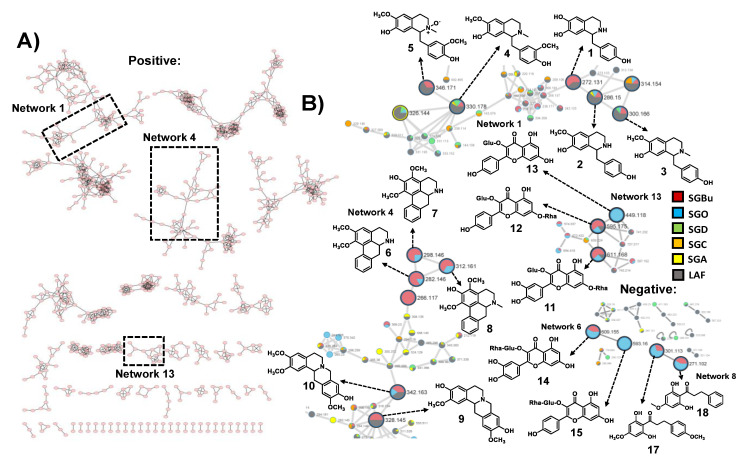
(**A**) Molecular networking through LC-HRMS/MS data in positive ionization mode of SGBu and bioactive CPC fractions. (**B**) Focusing on the networks with the annotated compounds in bioactive fractions through LC-HRMS/MS data in positive and negative ionization modes. Crude *n*-butanol extract (SGBu) 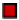; the most active fractions (SGO) 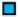, (SGD) 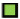, (SGC) 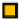 and (SGA) 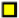; the least active fractions (LAF) 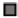, which were SGB, SGE, SGF, SGG, SGH, SGI and SGK.

Untargeted LC-HRMS/MS analyses in positive and negative ionization modes were performed in the CPC fractions to characterize their metabolites. The resulting spectral data of fractions with cell viability above 80% and antiviral activity against influenza virus (Figure 1) were analyzed using the GNPS platform to generate molecular networks. For this, molecular network creation was based on mass spectrometry data analysis of the SGBu extract (G1), the most active fractions (enumerated as decreasing activity results) SGO (G2), SGD (G3), SGC (G4), and SGA (G5) (Supplementary Figure S6), and the least active fractions (LAF), which were SGB, SGE, SGF, SGG, SGH, SGI and SGK, all placed into the same group (G6). After data processing, a total of 30 networks were obtained, comprising 592 nodes for positive mode and 8 networks with 23 nodes for negative mode (Figure 2). Several hits were obtained, all of them checked manually by MS/MS data interpretation to validate the identifications. Most of the annotated compounds in the CPC fractions belong to the isoquinoline alkaloid class, in addition to other compounds, such as flavonoid glycosides and dihydrochalcones.

Among the most active fractions, all displayed alkaloids within their composition. SGA, SGC, and SGD, collected during elution step within the first 43 min of process, are composed of coclaurine **2** and reticuline **4**, in addition to *N*-methylcoclaurine **3** for SGD. The molecular network 1 displays the nodes of **2** (*m/z* 286.1444), **3** (*m/z* 300.1593), and **4** (*m/z* 330.1720), whereas other nodes with *m/z* 272.1283 and *m/z* 346.1655, which can correspond to demethyl-coclaurine **1** and reticuline *N*-oxide **5**, were annotated in LAF only (Table 1); (Figure 2). These attributions were proposed based on diagnostic fragment ions and neutral losses characteristic for benzylisoquinoline alkaloids as previously described [32].

Moreover, other nodes in network 4 (*m/z* 328.1564, **9** and *m/z* 342.1704, **10**) are consistent with the structures of alkaloids. However, their fragmentation indicates that they are tetrahydroprotoberberine derivatives, especially due to the appearance of the diagnostic fragment ion of *m/z* 178, formed via a retro-Diels-Alder ring opening, typical in MS/MS spectra of this type of alkaloid skeleton [32]. Therefore, these compounds were annotated as stepholidine **9** and isocorypalmine **10**. All these compounds were eluted and detected in LAF. In addition, compound **10** also appeared at SGO active fraction (Table 1); (Figure 2).

On the other hand, in fractions collected in the extrusion step with the change from mobile phase to upper organic phase, aporphine alkaloids, *O*-glycosylated flavonoids, and dihydrochalcones eluted in SGO fraction appeared in networks 4, 6, 8, and 13 (Table 1); (Figure 2). Three aporphine derivatives grouped at network 4 were annotated based on their fragmentation pattern. As expected for aporphines, the initial losses of 17 u were observed, and intense corresponding fragments were formed. Moreover, some fragmentation features suggested adjacent methoxy and hydroxy-groups in compound **7** (CH_3_OH followed by CO losses, −32 u and −28 u) and adjacent methoxy groups in compounds **6** and **8** (CH_3_ and CH_3_O group radical losses, −15 u and −31 u, respectively) [32,33]. Thus, nornuciferine **6**, isopiline **7**, and *O*-methylisopiline **8** were annotated.

Additionally, the ions at *m/z* 611.1639 **11**, *m/z* 595.1705 **12**, and *m/z* 449.1096 **13** in the positive mode, and *m/z* 609.1524 **14** and *m/z* 593.1579 **15** in the negative mode, showed neutral sugar losses of 146 u (deoxyhexosides) and 162 u (hexosides) on their MS/MS spectra. Compounds **11** and **14**, displayed fragments consistent with the aglycone quercetin, while compounds **12**, **13**, and **15** displayed those of kaempferol as their aglycone. Thus **11**, **12**, **13**, **14**, and **15** were annotated as quercetin 3-*O*-rhamnoside-7-*O*-glucoside or quercetin 3-*O*-glucoside-7-*O*-rhamnoside, kaempferol 3-*O*-glucoside-7-*O*-rhamnoside, kaempferol 3-*O*-glucoside, quercetin-3-*O*-rutinoside (rutin), and kaempferol 3-*O*-rutinoside, respectively [34,35,36]. In addition, the MS/MS spectra for the compounds of *m/z* 301.1104 **16** and *m/z* 271.1012 **17** observed at the negative mode displayed diagnostic ions of A-type fragment [^1^A-CH_3_]^−^, common for dihydrochalcones [37]. Therefore, these compounds **16** and **17** were assigned as 2′,6′-dihydroxy-4,4′-dimethoxydihydrochalcone and 2′,6′-dihydroxy-4′-methoxy-dihydrochalcone, respectively.

The SGJ, SGL, SGM, SGN, SGP, SGQ, and SGR fractions, with cell viability values below 50%, also had their chemical profile investigated by LC-HRMS/MS. The ion of *m/z* 328.1564 [M + H]^+^ corresponding to stepholidine **9** (Supplementary Figure S15) was common for SGJ, SGL, SGM, SGN, SGP, and SGQ. Furthermore, the MS/MS spectrum of compound of *m/z* 314.1792 [M]^+^ in the SGP, SGQ and SGR showed characteristic fragment ions for magnocurarine (Supplementary Figure S24) [38]. In addition to alkaloids in SGJ, SGL, and SGQ, flavonoids were observed with galloyl units and *O*-triglycosylated flavonoid. The ion of *m/z* 727.2089 [M + H]^+^ in SGJ and SGL showed, in MS/MS spectrum, fragments of *m/z* 449 and *m/z* 287, which likely result from a loss of pentose, deoxyhexose, and hexose units, corresponding to kaempferol-3-*O*-hexose-*O*-deoxyhexose-*O*-pentoside (Supplementary Figure S25) [39]. In SGQ, the ions of *m/z* 585.2190 [M − H]^−^ and *m/z* 615.2335 [M − H]^−^ produced the product ions of *m/z* 301 [M − H-152-132]^−^ (Supplementary Figure S26) and *m/z* 301 [M − H-152-162]^−^ (Supplementary Figure S27), indicating a galloyl-pentoside and galloyl-hexoside moiety losses, respectively. Through this fragmentation pattern, these compounds were annotated as quercetin-3-*O*-(2′’-*O*-galloyl)-pentoside and quercetin-3-*O*-(6′’-*O*-galloyl)-β-galactopyranoside [40,41].

## 3. Materials and Methods

### 3.1. Solvents and Chemical Reagents

Analytical grade solvents from Tedia^®^ Brazil (Tedia, Rio de Janeiro, Brazil) were used for the preparation of extracts. CPC fractionation was performed with ultrapure water (18.2 MΩ-cm) prepared with a PURELAB^®^ Classic system (ELGA, Woodridge, IL, USA), and analytical grade solvents from Tedia^®^ Brazil (Rio de Janeiro, Brazil). LC-HRMS/MS analyses were carried out using analytical grade solvents from Tedia^®^ Brazil (Rio de Janeiro, Brazil), ammonium formate (CAS number: 540-69-2) purchased from Sigma-Aldrich^®^, formic acid from Vetec Química Fina^®^, LTDA (Rio de Janeiro, Brazil), and ultrapure water (18.2 MΩ-cm).

### 3.2. Plant Material and Extracts Preparation

Leaves of *Siparuna glycycarpa* (Ducke) S.S. Renner & Hausner were collected at Reserva Adolpho Ducke, Manaus, Brazil, in August 2015 and deposited at Instituto Nacional de Pesquisas da Amazônia (INPA) herbarium (Manaus, Brazil) under the registration INPA 269732. This research was authorized by the Directing Council of Genetic Heritage (Conselho de Gestão do Patrimônio Genético, CGEN) under the authorization A3C04CB. The leaves were dried and ground (101.97 g) and were extracted by percolation with ethanol 96° GL. Part of the crude ethanol extract (14.1 g, 13.8% *w*/*w*) was fractionated by liquid–liquid extraction between water–methanol 7:3 (*v*/*v*) and organic solvents affording *n*-hexane (2.80 g), dichloromethane (2.20 g), ethyl acetate (2.33 g), and *n*-butanol (2.18 g) extracts.

### 3.3. Thin Layer Chromatography (TLC)

The preliminary analyses of the *n*-butanol extract, solvent system selection tests, and CPC fractions were performed on silica gel TLC plates (AL 60 F254 20 cm × 20 cm MERCK^®^, Darmstadt, Germany). The TLC plates were eluted with the organic phase of *n*-butanol–acetic acid–water (BAW) (8:2:10, *v/v*), and the results were visualized under ultraviolet light (UV) (Spectroline CL-80 model) at 254 nm and 365 nm.

### 3.4. Centrifugal Partition Chromatography (CPC)

#### 3.4.1. Apparatus

The fractionation step was performed on a CPC Lab System (Gilson, Middleton, WI, USA) that combines a PLC purification system (model 2050) with a CPC 250. The column has a total volume of 250 mL and can be used with a pressure up to 100 bar, flow rate up to 15 mL·min^−1^, and a maximum rotation speed of 3000 rpm. The PLC features a fraction collector and a built-in detector that measures the absorbance and sends the chromatogram to the onboard control software, Gilson Glider Prep (GGP) software. The dissolved sample was injected through the sample loop of 10 mL.

#### 3.4.2. Solvent System Selection

The solvent system composed of *n*-butanol–methanol–water was selected, and different volume ratios (10:0:10, *v/v*), (9:1:10, *v/v*), and (8:2:10, *v/v*) were screened. The *n*-butanol extract (2 mg) was dissolved in test tubes containing 2 mL of each phase of the solvent systems and were shaken to allow compounds to partition between the two phases (Supplementary Figure S1).

#### 3.4.3. Solvent System and Sample Preparation

The selected solvent system *n*-butanol-methanol-water (9:1:10, *v/v*) was thoroughly equilibrated and the two separated phases were degassed by ultra-sonication for 10 min. The sample solution was prepared dissolving 500 mg of the *n*-butanol extract in 10 mL of the solvent system (1:1, *v/v*).

#### 3.4.4. CPC Fractionation Procedure

The fractionations were performed with the lower aqueous phase as mobile phase and upper organic phase as stationary phase (descending mode). The CPC column was first entirely filled with the upper organic stationary phase and then the lower aqueous mobile phase was pumped through the column at 6 mL·min^−1^ flow rate in the descending mode until hydrostatic equilibrium, when the sample was injected into the CPC column. Three fractionations of the *n*-butanol extract were performed with different rotation speeds: 1000 and 1300 rpm and the UV monitoring at different wavelength (254 nm, 365 nm and 200–400 nm-full scan). In the first and second fractionations 47 tubes of 12 mL were collected (22 tubes with classic elution step and 25 tubes with extrusion step). For the last fractionation, the local minimum function was applied for the fraction collection step. A total of 167 fractions were collected: 94 fractions in the elution step, corresponding to 250 mL mobile phase (1.0 column-volume) and 73 fractions in the extrusion step, corresponding to 302.2 mL (1.2 column-volume) of pumped upper phase.

### 3.5. CPC Fractions Preparation for Antiviral and Cytotoxicity Assays

Dried fractions and *S. glycycarpa n*-butanol extract were dissolved in dimethyl sulfoxide (DMSO) to a final concentration of 100 mg·mL^−1^ (stock solution) for the in vitro tests. Cell culture medium was used to dilute fractions, and *n*-butanol extract and oseltamivir carboxylate (OST-car) were used to obtain the assays concentrations. For all assays, DMSO did not exceed 1% (*v*/*v*).

### 3.6. Cells and Viruses

Madin-Darby Canine Kidney (MDCK) cells were cultured in Dulbecco’s Modified Eagle Medium (DMEM; Life Technologies, Grand Island, NY, USA) supplemented with 10% fetal bovine serum (FBS; HyClone, Logan, UT, USA), 100 U/mL penicillin, and 100 mg/mL streptomycin (Sigma-Aldrich, Burlington, MA, USA) and maintained at 37 °C in a 5% CO_2_ atmosphere.

Cell infection assays were performed with influenza A (IAV) strain (A/Michigan/45/2015), an influenza A(H1N1)pdm09 virus, propagated in MDCK cells. IAV was grown and titrated according to the World Health Organization (WHO) manual for the laboratory diagnosis and virological surveillance of influenza [42]. Viral stocks were aliquoted and stored at −70 °C for further studies.

The cell and virus were kindly provided by the Centers for Disease Control and Prevention (CDC, Atlanta, GA, USA) and Influenza Reagent Resources (IRR).

### 3.7. Cell Viability (Cytotoxicity Assay)

Monolayers of 2 × 10^4^ MDCK cells in 96-well plates were treated with 100 µg/mL of *n*-butanol extract from *S. glycycarpa* and fractions for 3 days. OST-car at 0.05 µM was used as control. The culture medium was removed and 5 mg/mL of (3-[4,5-dimethylthiazol-2-yl]-2,5 diphenyl tetrazolium bromide) (MTT) in phosphate-buffered saline (PBS) 1x was added. Cells were incubated for 2 h at 37 °C. A 10% *w/v* sodium dodecyl sulfate (SDS) solution was added followed by another 2 h-incubation period. The plate was read in a spectrophotometer at 570 nm.

### 3.8. Influenza Replication Inhibition Assay

Monolayers of MDCK cells in 96-well plates (2 × 10^4^ cells/well) were infected with influenza A/H1N1 virus at a multiplicity of infection (MOI) of 0.01 for 1 h at 37 °C. The inoculum was removed, and cells were treated with 25 or 100 µg/mL of *n*-butanol extract from *S. glycycarpa* and fractions for 24 h at 37 °C. OST-car at 0.0125 or 0.05 was used as control. These concentrations are equivalent to EC_50_ and 4 times the EC_50_ values for *S. glycycarpa n*-butanol extract and OST-car [8]. After the incubation, the culture supernatant was collected, and viruses were quantified by viral neuraminidase activity.

### 3.9. Neuraminidase Activity

The activity of viral neuraminidase (NA) was measured in the viruses present in the supernatants harvested from the influenza replication inhibition assays using the NA-Star kit (Applied Biosystems, Foster City, CA, USA), according to the manufacturer’s instructions. Briefly, culture supernatants were incubated for 30 min with a chemiluminescent neuraminidase substrate at room temperature, and then an accelerator solution was added triggering light emission from the cleaved substrate. The chemiluminescent signal was read in the luminometer.

### 3.10. Statistical Analysis

All the antiviral and cytotoxicity tests were performed at least three times, and the results are displayed as the mean ± standard error, calculated using Excel 2010 for Windows software (Microsoft).

### 3.11. LC-HRMS/MS Analysis

Analysis of the CPC fractions were performed on an ultra-fast liquid chromatography (UFLC) apparatus (Shimadzu), coupled to a MicrOTOF-QIII-MS (Bruker Daltonics, Inc., Billerica, MA, USA) mass spectrometer with ESI ionization source. The samples (0.5 mg) were dissolved in 2 mL of methanol, and 3 µL was injected on a column Acquity UPLC^®^ BEH C18 (2.1 mm × 150 mm internal diameter (i.d.); 1.7 µm particle size; Waters, Dublin, Ireland) heated at 40 °C. The mobile phase consisted of water–5 mM ammonium formate–0.1% formic acid (A) and methanol (B). The gradient conditions were set as B = 50% at 0 min, B = 99% at 10 min, B = 100% at 30 min and remains at 100% until 32 min, B = 50% at 35 min and remains at 50% until 45 min at a flow rate of 0.2 mL·min^−1^. The source temperature was set at 200 °C, 8 L·min^−1^ dry gas (nitrogen) flow rate and 4 bar nebulizer gas pressure (nitrogen). Data were acquired in positive and negative ionization modes in the mass range of *m/z* 50–1200, and collision-induced dissociation (CID) cell energy was set to 20 eV. The chromatograms and mass spectra were processed in Bruker Daltonics ESI Compass Data Analysis software version 5.0 SR1 (Bruker Daltonics, Inc., Billerica, MA, USA).

### 3.12. Global Natural Products Social Molecular Networking (GNPS)

Product ion spectra arising from the LC-HRMS/MS analysis of the *S. glycycarpa n*-butanol extract and its bioactive fractions were analyzed and organized in molecular networks by using the GNPS platform (http://gnps.ucsd.edu, accessed on 21 October 2021) [43]. The MS/MS data were converted to the mzXML format with MS-Convert [44] and then uploaded on the GNPS Web platform. Parameters for molecular network generation were set as follows: the precursor ion mass tolerance of 0.05 Da, product ion tolerance of 0.5 Da, and fragment ions below 10 counts were removed from the MS/MS spectra. Molecular networks were generated using four minimum matched peaks and a cosine score of 0.65. Data were visualized using Cytoscape 3.7.0 software. Annotation of chemical compounds was performed by manual interpretation of MS/MS spectra in comparison with the databases inside GNPS. The MS/MS molecular network is accessible at the GNPS Web site with the following links: https://gnps.ucsd.edu/ProteoSAFe/status.jsp?task=daf5b5b0355e4862b3f163d3d62052cc, accessed on 21 October 2021, and https://gnps.ucsd.edu/ProteoSAFe/status.jsp?task=9dc29a10767344f5902b709fdb440722, accessed on 21 October 2021.

## 4. Conclusions

The search for new anti-influenza agents is essential for public health. In this study, the application of CPC enabled the bioassay-guided fractionation of *S. glycycarpa n*-butanol extract by using a solvent system that failed stationary phase retention on HSCCC, therefore acquiring bioactive fractions. With the use of a local minimum function tool, it was possible to improve resolution during chromatographic separation. Different compounds were annotated through dereplication tools, while changing the mobile phase nature made it possible to obtain a distinct chemical profile of bioactive fractions composition. Furthermore, different compounds such as flavonoids with galloyl units and *O*-triglycosylated flavonoid were annotated in fractions with cell viability values below 50%. Benzylisoquinolinic and aporphine alkaloids were annotated in the most bioactive fractions, whereas tetrahydroprotoberberines were at the least active ones. Moreover, glycosylated flavonoids derived from quercetin and kaempferol and dihydrochalcones were also annotated in one of the most active fractions, suggesting this group of compounds, along with the described alkaloids, were responsible for antiviral activity. Compound isolation is the aim of future research in order to assay their individual antiviral potential.

## Figures and Tables

**Table 1:** LC-HRMS/MS data of annotated compounds in CPC bioactive fractions (MS/MS spectra in Supplementary Figures S7–S23).

CPC Fractions	[M + H]^+^ (*m/z*)	[M − H]^−^ (*m/z*)	Molecular Formula	Error (ppm)	MS/MS (MS^2^)	Proposed Compound
**Network 1** **(Positive Ionization Mode Data)**						
LAF	272.1283	-	C_16_H_17_NO_3_	−1.10	255.10, 237.08, 223.07, 209.09, 194.06	Demethyl-coclaurine **1**
All fractions	286.1444	-	C_17_H_19_NO_3_	0.35	269.12, 237.09, 209.09, 194.07, 178.08, 115.05, 107.05	Coclaurine **2**
SGO, SGD, LAF	300.1593	-	C_18_H_21_NO_3_	−1.99	269.11, 257.11, 237.09, 225.08, 209.09, 194.07, 181.06, 107.04	*N*-methylcoclaurine **3**
All fractions	330.1720	-	C_19_H_23_NO_4_	4.54	299.13, 265.08, 207.0793, 192.10, 178.08, 163.06, 137.06	Reticuline **4**
LAF	346.1655	-	C_19_H_23_NO_5_	0.28	329.16, 312.12, 299.13, 286.11, 267.09, 238.08, 185.08, 137.06, 115.05, 91.05	Reticuline *N*-oxide **5**
**Network 4** **(Positive Ionization Mode Data)**						
SGO	282.1475	-	C_18_H_19_NO_2_	−6.73	265.12, 250.10, 234.10, 219.07, 207.08, 189.07, 179.08	Nornuciferine **6**
SGO	298.1438	-	C_18_H_19_NO_3_	−1.67	281.11, 266.09, 250.09, 233.06, 221.09, 205.06, 189.06, 178.07	Isopiline **7**
SGO	312.1595	-	C_19_H_21_NO_3_	−1.28	295.13, 280.10, 264.11, 249.08, 234.06, 219.07	*O*-Methylisopiline **8**
LAF	328.1564	-	C_19_H_21_NO_4_	4.87	313.13, 192.10, 178.08	Stepholidine **9**
SGO, LAF	342.1704	-	C_20_H_23_NO_4_	−0.29	324.19, 312.12, 297.11, 194.08, 178.08, 163.06	Isocorypalmine **10**
**Network 13** **(Positive Ionization Mode Data)**						
SGO, LAF	611.1639	-	C_27_H_30_O_16_	4.41	465.10, 303.05	Quercetin-3-*O*-rhamnoside-7-*O*-glucoside **11** Quercetin-3-*O*-glucoside-7-*O*-rhamnoside **11**
SGO, LAF	595.1705	-	C_27_H_30_O_15_	7.05	449.11, 287.05	Kaempferol-3-*O*-glucoside-7-*O*-rhamnoside **12**
SGO	449.1096	-	C_21_H_20_O_11_	2.67	287.05, 249.14, 227.17, 100.11	Kaempferol 3-*O*-glucoside **13**
**Networks 6 and 8** **(Negative Ionization Mode Data)**						
SGO	-	609.1524	C_27_H_30_O_16_	11.0	300.03, 271.02, 243.03, 199.04, 151.00, 148.01, 108.02	Quercetin-3-*O*-rutinoside (Rutin) **14**
SGO	-	593.1579	C_27_H_30_O_15_	−12.0	285.04, 255.03, 227.04, 211.0433, 183.05, 107.01	Kaempferol-3-*O*-rutinoside **15**
SGO	-	301.1104	C_17_H_18_O_5_	9.30	253.05, 225.05, 188.05, 165.02, 152.01, 124.01	2′,6′-Dihydroxy-4,4′-dimethoxydihydrochalcone **16**
SGO	-	271.1012	C_16_H_16_O_4_	15.5	238.06, 210.07, 184.00, 165.02, 152.01, 124.01	2′,6′-Dihydroxy-4′-methoxy-dihydrochalcone **17**

## Data Availability

Not applicable.

## Supplementary Materials

The following are available online, Figure S1: TLC results for solvent system selection with the test tube partitioning test. Tested solvent systems: *n*-butanol–methanol–water (1) 10:0:10, (2) 9:1:10, (3) 8:2:10 (*v*/*v*). (UP) upper phase and (LP) lower phase. The TLC plate was eluted with the organic phase of *n*-butanol–acetic acid–water (8:2:10, *v*/*v*) solvent system followed by visualized under UV light (254 nm (A) and 365 nm (B)). Figure S2: (A) CPC-UV chromatogram of the *n*-butanol extract fractionation at 1000 rpm rotation ( 254 nm;  365 nm;  200–400 nm). (B) TLC analysis of CPC fractions pooled according to chemical and chromatographic similarities. Figure S3: (A) CPC-UV chromatogram of the *n*-butanol extract fractionation at 1300 rpm rotation ( 254 nm;  365 nm;  200–400 nm). (B) CPC fractions by TLC analysis. Figure S4: Ultraviolet spectra of selected fractions acquired by CPC fractionation at 1300 rpm rotation. Figure S5: (A) CPC-UV chromatogram of the *n*-butanol extract fractionation at 1300 rpm rotation and local minimum function ( 254 nm;  365 nm;  200–400 nm). (B) CPC fractions by TLC analysis. Figure S6: (A) SGO, (B) SGD, (C) SGA, (D) SGC LC-HRMS/MS analysis in positive ionization mode. (E) SGO LC-HRMS/MS analysis in negative ionization mode. Figure S7: (A) MS spectrum with *m/z* 272.1283 [M + H]^+^ and (B) MS^2^ spectrum of demethyl-coclaurine 1. Figure S8: (A) MS spectrum with *m/z* 286.1444 [M + H]^+^ and (B) MS^2^ spectrum of coclaurine 2. Figure S9: (A) MS spectrum with *m/z* 300.1593 [M + H]^+^ and (B) MS^2^ spectrum of *N*-methylcoclaurine 3. Figure S10: (A) MS spectrum with *m/z* 330.1720 [M + H]^+^ and (B) MS^2^ spectrum of reticuline 4. Figure S11: (A) MS spectrum with *m/z* 346.1655 [M + H]^+^ and (B) MS^2^ spectrum of reticuline *N*-oxide 5. Figure S12: (A) MS spectrum with *m/z* 282.1475 [M + H]^+^ and (B) MS^2^ spectrum of nornuciferine 6. Figure S13: (A) MS spectrum with *m/z* 298.1438 [M + H]^+^ and (B) MS^2^ spectrum of isopiline 7. Figure S14: (A) MS spectrum with *m/z* 312.1595 [M + H]^+^ and (B) MS^2^ spectrum of *O*-methylisopiline 8. Figure S15: (A) MS spectrum with *m/z* 328.1564 [M + H]^+^ and (B) MS^2^ spectrum of stepholidine 9. Figure S16: (A) MS spectrum with *m/z* 342.1704 [M + H]^+^ and (B) MS^2^ spectrum of isocorypalmine 10. Figure S17: (A) MS spectrum with *m/z* 611.1639 [M + H]^+^ and (B) MS^2^ spectrum of quercetin-3-*O*-rhamnoside-7-*O*-glucoside or quercetin-3-*O*-glucoside-7-*O*-rhamnoside 11. Figure S18: (A) MS spectrum with *m/z* 595.1705 [M + H]^+^ and (B) MS^2^ spectrum of kaempferol-3-*O*-glucoside-7-*O*-rhamnoside 12. Figure S19: (A) MS spectrum with *m/z* 449.1096 [M + H]^+^ and (B) MS^2^ spectrum of kaempferol 3-*O*-glucoside 13. Figure S20: (A) MS spectrum with *m/z* 609.1524 [M − H]^−^ and (B) MS^2^ spectrum of quercetin-3-*O*-rutinoside (Rutin) 14. Figure S21: (A) MS spectrum with *m/z* 593.1579 [M − H]^−^ and (B) MS^2^ spectrum of kaempferol-3-*O*-rutinoside 15. Figure S22: (A) MS spectrum with *m/z* 301.1104 [M − H]^−^ and (B) MS^2^ spectrum of 2′,6′-dihydroxy-4,4′-dimethoxydihydrochalcone 16. Figure S23: (A) MS spectrum with *m/z* 271.1012 [M − H]^−^ and (B) MS^2^ spectrum of 2′,6′-dihydroxy-4′-methoxy-dihydrochalcone 17. Figure S24. MS^2^ spectrum of magnocurarine *m/z* 314.1792 [M]^+^. Figure S25. MS^2^ spectrum of kaempferol-3-*O*-hexose-*O*-deoxyhexose-*O*-pentoside *m/z* 727.2089 [M + H]^+^. Figure S26. MS^2^ spectrum of quercetin-3-*O*-(2′’-*O*-galloyl)-pentoside *m/z* 585.2190 [M − H]^−^. Figure S27. MS^2^ spectrum of quercetin-3-*O*-(6′’-*O*-galloyl)-β-galactopyranoside *m/z* 615.2335 [M − H]^−^. Table S1: Pooled fractions from the *n*-butanol extract fractionation at 1300 rpm rotation and local minimum function (Supplementary Figure S5). Table S2: Cell viability of pooled fractions from the *n*-butanol extract fractionation by CPC. Table S3: Anti-influenza activity of CPC pooled fractions with cell viability above 80%.

## Sample Availability

Samples are not available.
