# Supplementary material for: Bioassay-Guided Fractionation of Siparuna glycycarpa n-Butanol Extract with Inhibitory Activity against Influenza A(H1N1)pdm09 Virus by Centrifugal Partition Chromatography (CPC)
Source: Molecules. 2022 Jan 8;27(2):399. doi: 10.3390/molecules27020399 (PMC8781433; doi:10.3390/molecules27020399)
Supplement: Supplementary file 1 [file molecules-27-00399-s001.zip › Supplementary material_FINAL.pdf]

Bioassay-Guided Fractionation of *Siparuna glycycarpa* *n*-butanol Extract with Inhibitory Activity Against Influenza A(H1N1)pdm09 Virus by Centrifugal Partition Chromatography (CPC)

Carla Monteiro Leal [1,2], Suzana Guimarães Leitão [3], Leonardo Luiz Oliveira de Mello [2], Isabel de Castro Rangel [2], Carlos Vinicius Azevedo da Silva [4], Milene Dias Miranda [5], Amanda Resende Tucci [5], Camilla Blanco de Assis [6,7], Carolina de Queiroz Sacramento [6,7], Natalia Fintelman-Rodrigues [6,7], Hector Henrique Ferreira Koolen [4], Boniek Gontijo Vaz [8], Rosineide Costa Simas [8] and Gilda Guimarães Leitão [2,*]

[1]Programa de Pós-Graduação em Biotecnologia Vegetal e Bioprocessos (PBV), Centro de Ciências da Saúde, Universidade Federal do Rio de Janeiro, Rio de Janeiro 21.941-902, Brazil; carlam.leal@yahoo.com.br

[2]Instituto de Pesquisas de Produtos Naturais, Centro de Ciências da Saúde, Universidade Federal do Rio de Janeiro, Rio de Janeiro 21.941-902, Brazil; leonardo_luiz_@outlook.com (L.L.O.d.M.); isabeldecastror@gmail.com (I.d.C.R.)

[3]Faculdade de Farmácia, Centro de Ciências da Saúde, Universidade Federal do Rio de Janeiro, Rio de Janeiro 21.941-902, Brazil; sgleitao@gmail.com

[4]Grupo de Pesquisas em Metabolômica e Espectrometria de Massas, Escola Superior de Ciências da Saúde, Universidade do Estado do Amazonas, Manaus 69.065-000, Brazil; cv25066@gmail.com (C.V.A.d.S.); hkoolen@uea.edu.br (H.H.F.K.)

[5]Laboratório de Vírus Respiratórios e do Sarampo, Instituto Oswaldo Cruz, Fundação Oswaldo Cruz, Rio de Janeiro 21.041-210, Brazil; milenediasmiranda@gmail.com (M.D.M.); biologa.t@gmail.com (A.R.T.)

[6]Laboratório de Imunofarmacologia, Instituto Oswaldo Cruz, Fundação Oswaldo Cruz, Rio de Janeiro 21.041-210, Brazil; camillablanco.a@gmail.com (C.B.d.A.); carol.qsacramento@gmail.com (C.d.Q.S.) nataliafintelman@gmail.com (N.F.-R.)

[7]Centro de Desenvolvimento Tecnológico em Saúde, Instituto Nacional de Ciência e Tecnologia de Gestão da Inovação em Doenças Negligenciadas, Fundação Oswaldo Cruz, Rio de Janeiro 21.041-210, Brazil

[8]Laboratório de Cromatografia e Espectrometria de Massas (LaCEM), Instituto de Química, Universidade Federal de Goiás, Goiânia 74.690-900, Brazil; boniek@gmail.com (B.G.V.); simas.rc@gmail.com (R.C.S.)

* Correspondence: ggleitao@ippn.ufrj.br

Supplementary Material

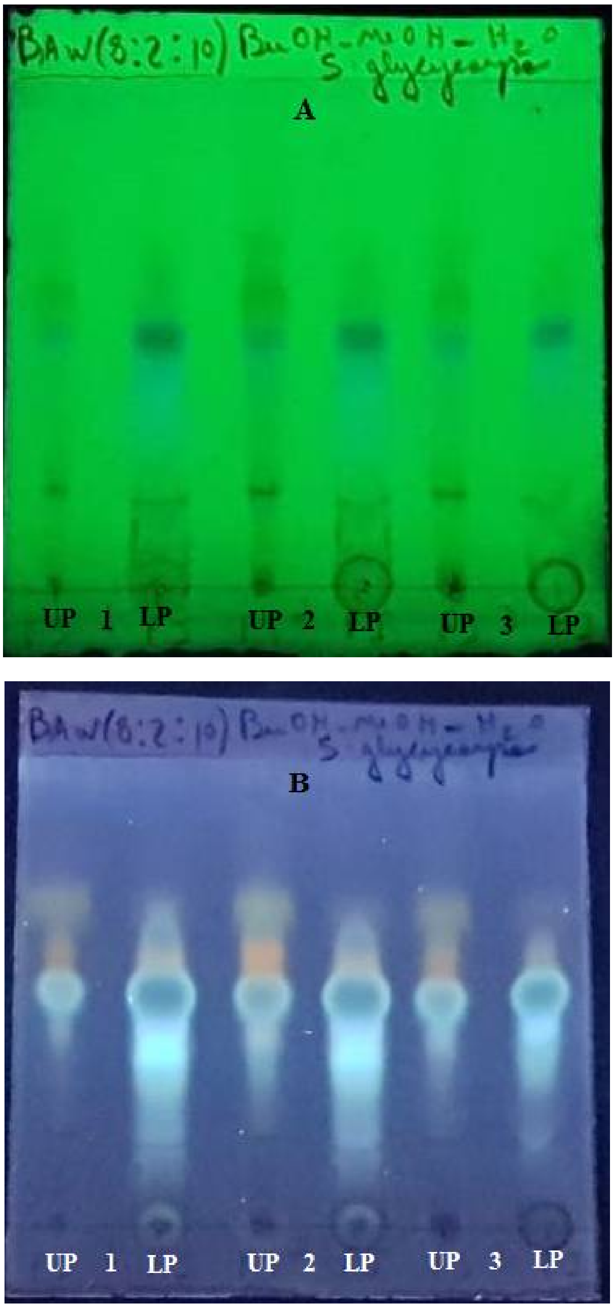

Supplementary Figure S1. TLC results for solvent system selection with the test tube partitioning test. Tested solvent systems: butanol-methanol-water (**1**) 10:0:10, (**2**) 9:1:10, (**3**) 8:2:10 (v/v). (**UP**) upper phase and (**LP**) lower phase. The TLC plate was eluted with the organic phase of butanol-acetic acid-water (8:2:10, v/v) solvent system followed by visualized under UV light (254 nm (**A**) and 365 nm (**B**)).

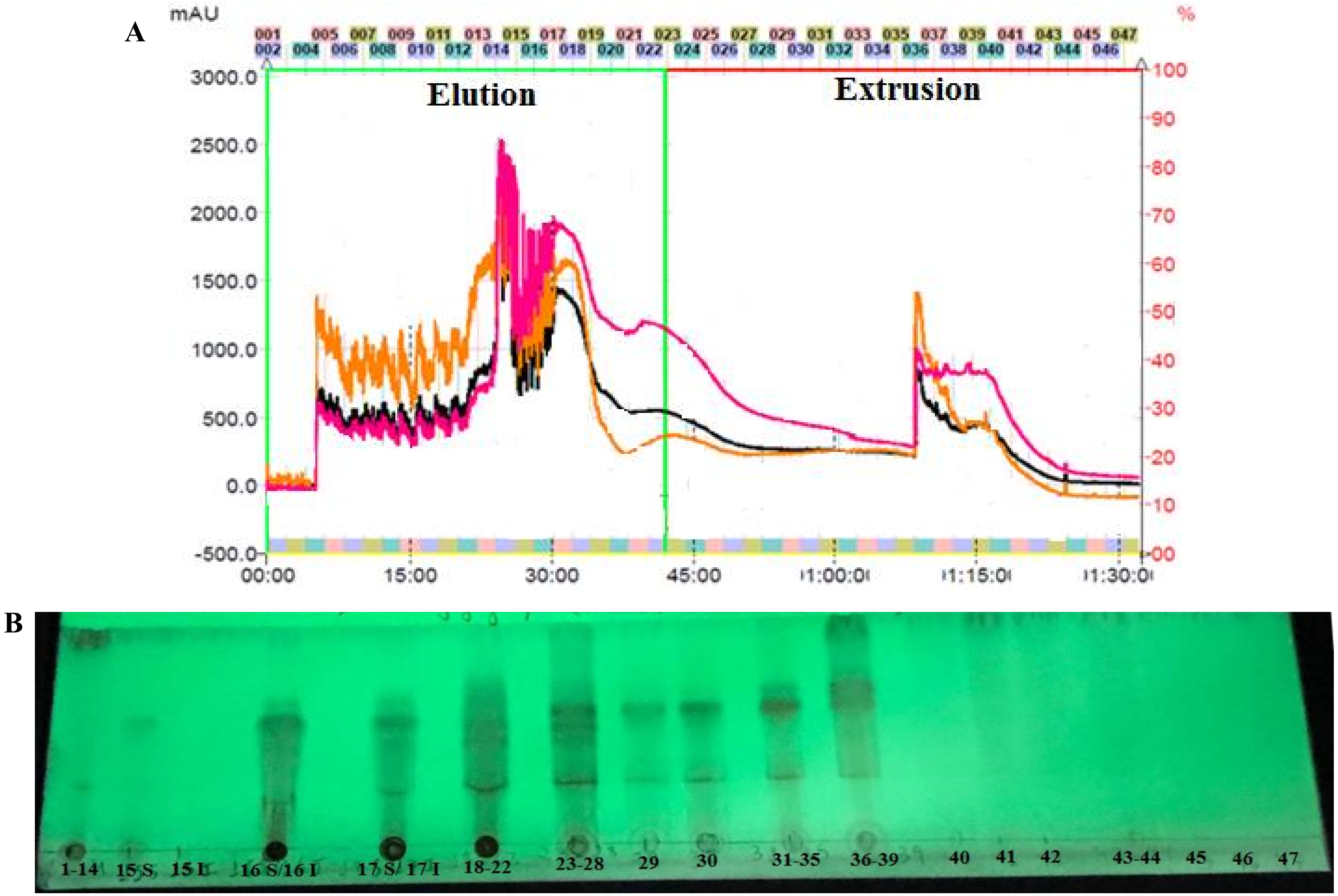

Supplementary Figure S2. (A) CPC-UV chromatogram of the *n*-butanol extract fractionation at 1000 rpm rotation (— 254 nm; — 365 nm; — 200-400 nm). **(B)** TLC analysis of CPC fractions pooled according to chemical and chromatographic similarities.

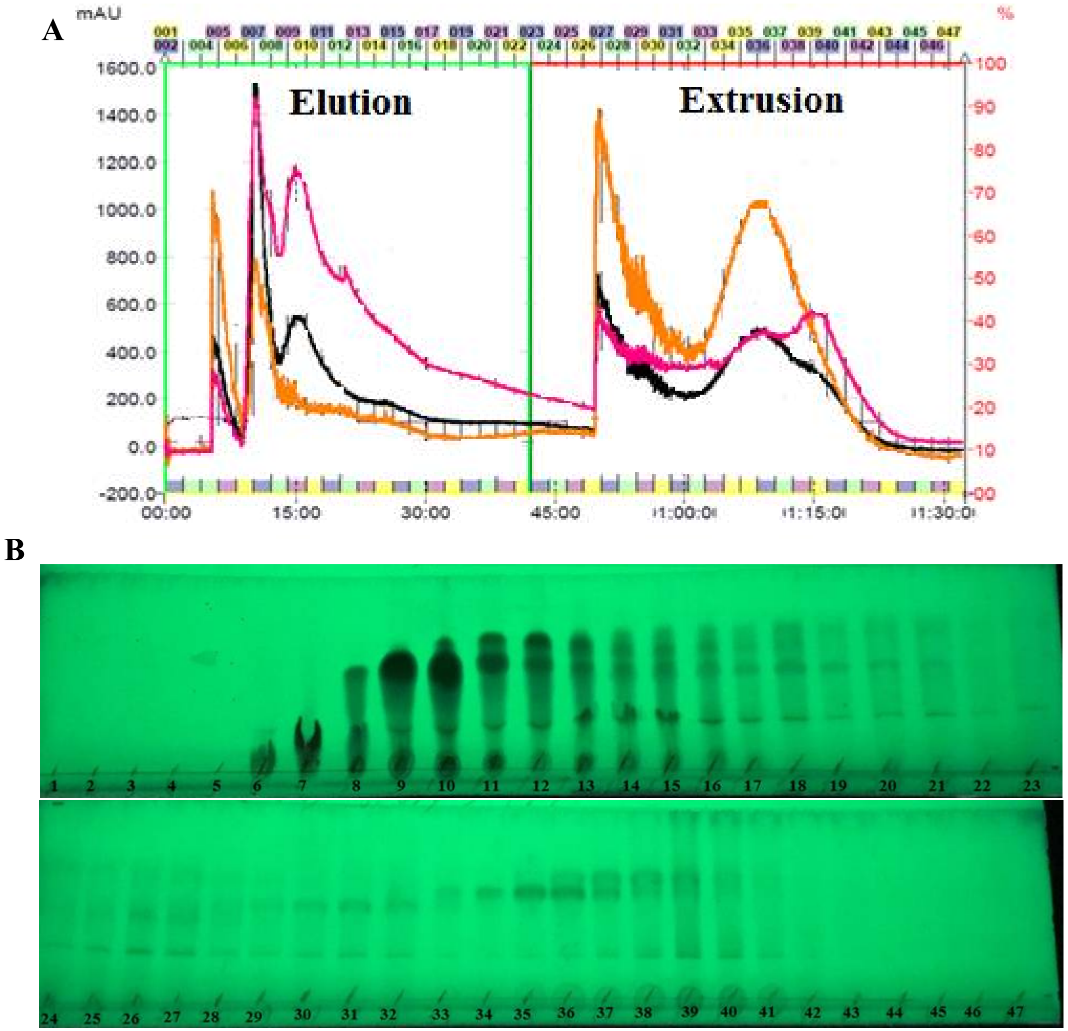

Supplementary Figure S3. (A) CPC-UV chromatogram of the *n*-butanol extract fractionation at 1300 rpm rotation (▬ 254 nm; ▬ 365 nm; ▬ 200-400 nm). **(B)** CPC fractions by TLC analysis.

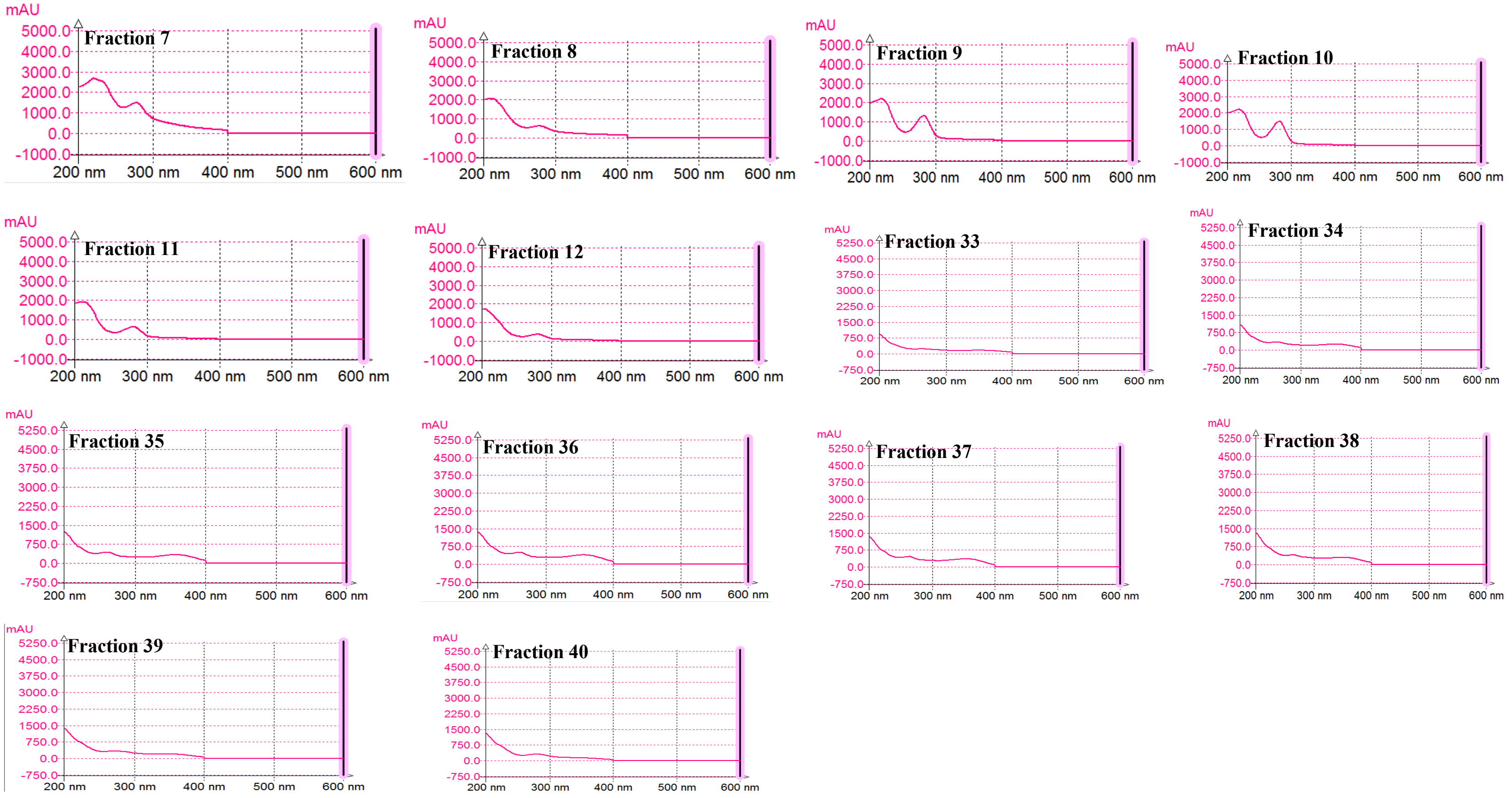

Supplementary Figure S4. Ultraviolet spectra of selected fractions acquired by CPC fractionation at 1300 rpm rotation.

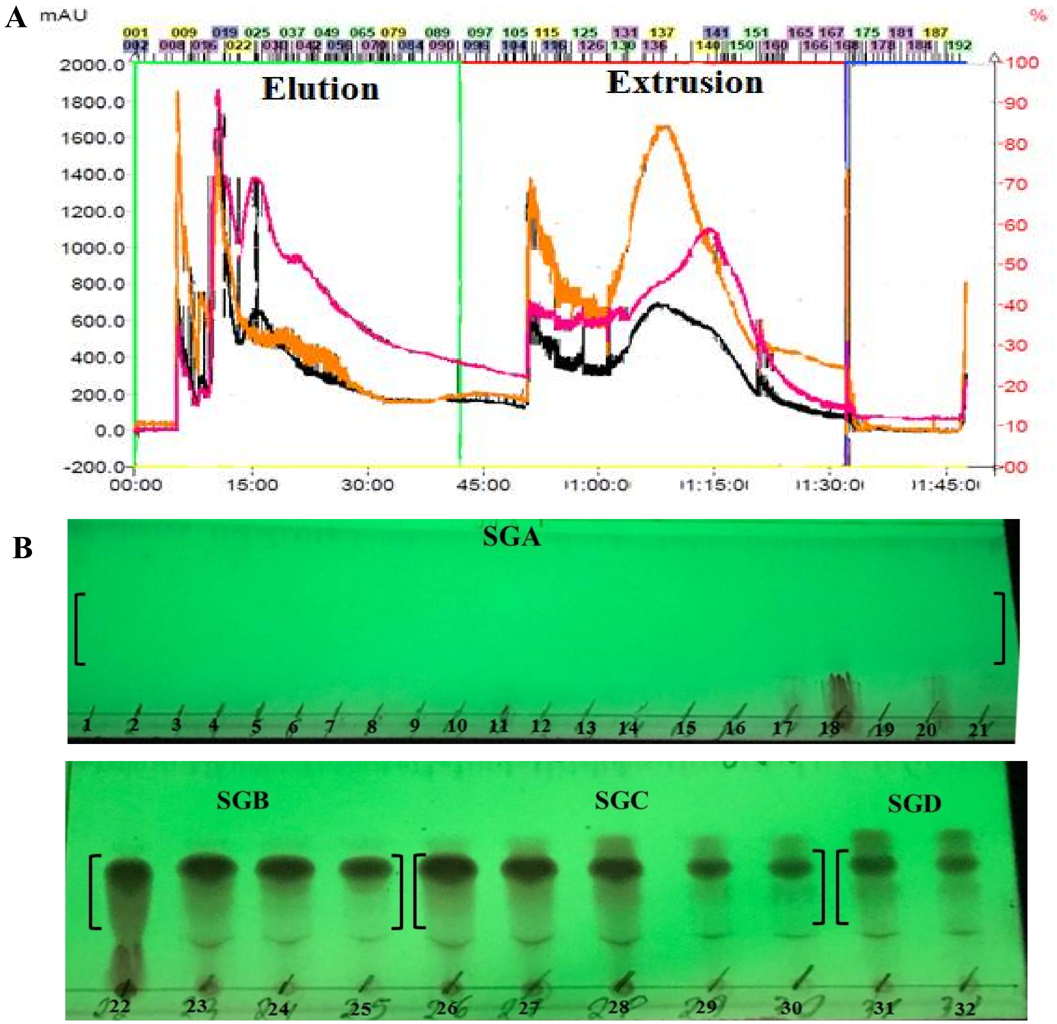

Supplementary Figure S5. (A) CPC-UV chromatogram of the *n*-butanol extract fractionation at 1300 rpm rotation and local minimum function (— 254 nm; — 365 nm; — 200-400 nm). **(B)** CPC fractions by TLC analysis.

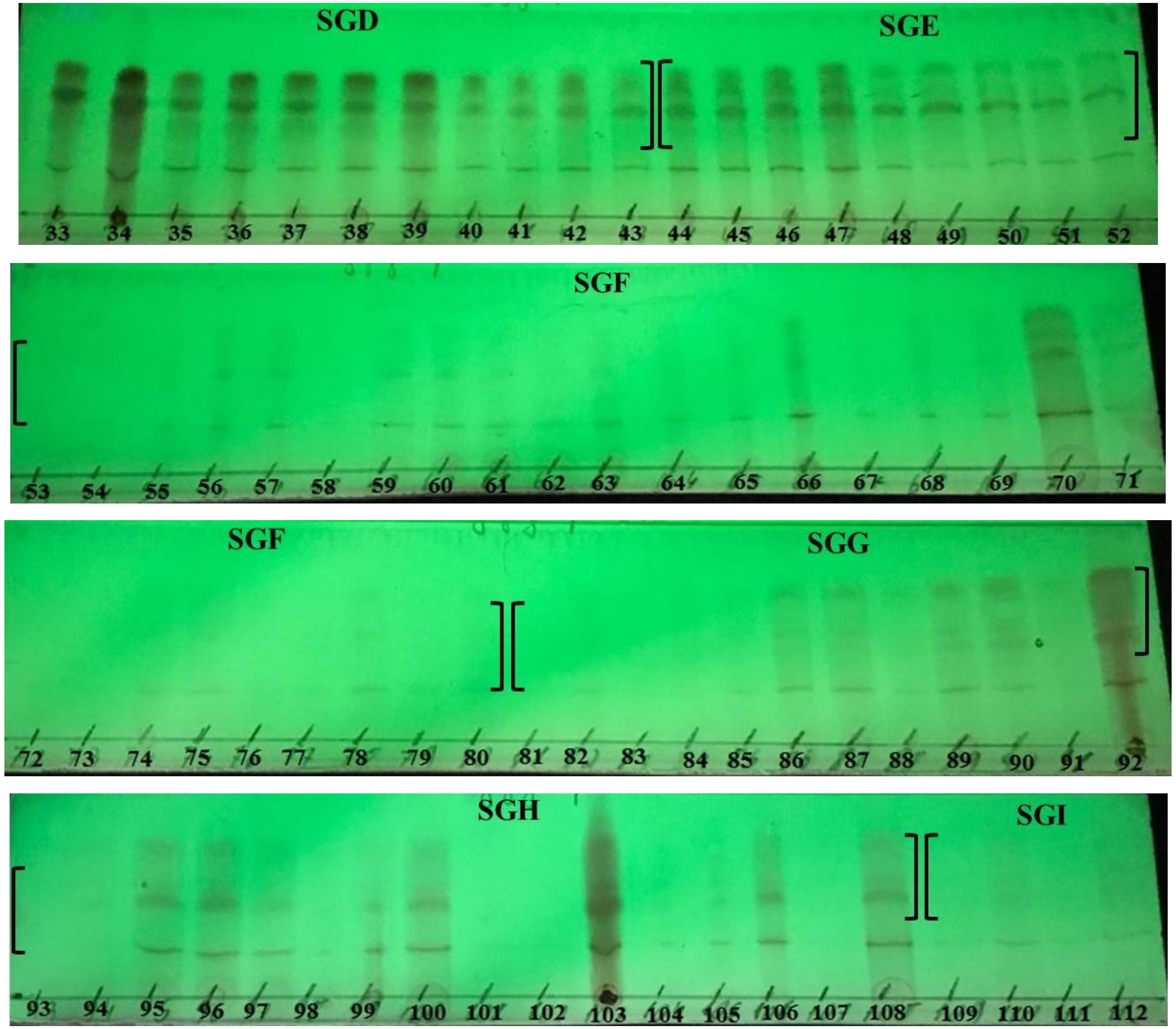

Supplementary Figure S5 continuation sequence. (B) CPC fractions by TLC analysis.

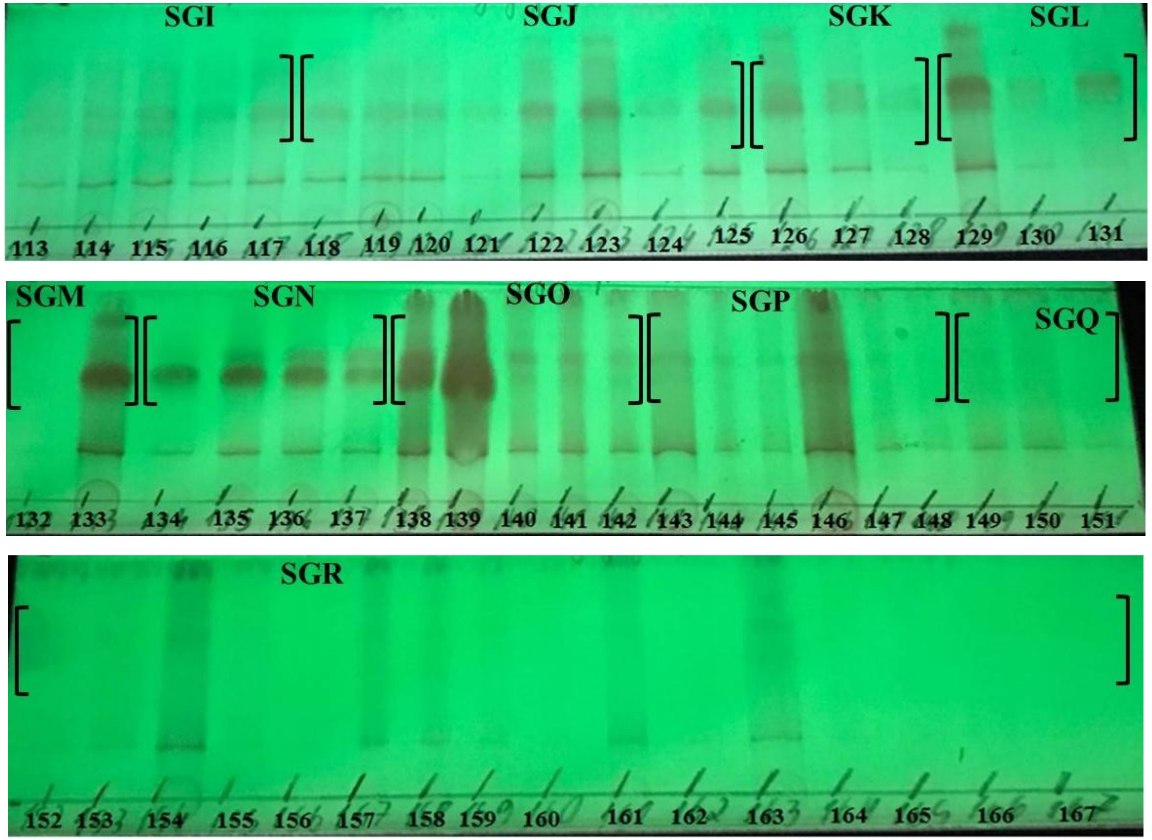

Supplementary Figure S5 continuation sequence. (B) CPC fractions by TLC analysis.

Supplementary Table S1. Pooled fractions from the *n*-butanol extract fractionation by CPC at 1300 rpm rotation and local minimum function (**Supplementary Figure S5**).

Pooled fractions	Sample code	Weight (mg)
1-21	SGA	80.6 mg
22-25	SGB	55.2 mg
26-30	SGC	23.4 mg
31-43	SGD	18 mg
44-52	SGE	6.3 mg
53-80	SGF	15 mg
81-92	SGG	16.8 mg
93-108	SGH	28.3 mg
109-117	SGI	13.5 mg
118-125	SGJ	22.9 mg
126-128	SGK	10.8 mg
129-131	SGL	15 mg
132-133	SGM	8.8 mg
134-137	SGN	14.1 mg
138-142	SGO	22.5 mg
143-148	SGP	21.8 mg
149-151	SGQ	10 mg
152-167	SGR	40.7 mg

Supplementary Table S2. Cell viability of pooled fractions from the *n*-butanol extract fractionation by CPC.

Fractions	Cell viability (%)* at 100 µg.mL^{-1}
SGA	81±5
SGB	88±1
SGC	100
SGD	100
SGE	100
SGF	83±5
SGG	86±6
SGH	91±2
SGI	80±5
SGJ	< 50
SGK	80±1
SGL	< 50
SGM	< 50
SGN	< 50
SGO	79±2
SGP	< 50

SGQ	< 50
SGR	< 50
SGBu	100
OST-car	87±3

SGBu, *n*-butanol extract from *Siparuna glycycarpa*.
OST-car, OST-carboxylate.
*DMSO was used as a cell control at 0.1 % v/v and the cell viability remained at least 95 % of cells exposed just to culture medium.

Supplementary Table S3. Anti-influenza activity of CPC pooled fractions with cell viability above 80%.

Fractions	Anti-influenza Screening (% of inhibition of viral replication)			
	24 hpi		48 hpi	
	**25 μg.mL^{-1}**	**100μg.mL^{-1}**	**25 μg.mL^{-1}**	**100μg.mL^{-1}**
SGA	37.0 ± 2.5	75.8 ± 3.9	27.6 ± 0.5	72.4 ± 0.6
SGB	15.8 ± 4.5	43.4 ± 4.4	6.2 ± 2.4	58.4 ± 1.7
SGC	36.4 ± 1.2	88.2 ± 2.3	27.9 ± 2.8	67.9 ± 6.1
SGD	59.2 ± 2.2	88.1 ± 1.4	39.1 ± 1.5	59.6 ± 7.9
SGE	15.9 ± 1.4	53.3 ± 6.9	NA	23.7 ± 4.9
SGF	16.3 ± 5.3	58.2 ± 3.7	17.1 ± 6.3	51.8 ± 7.1
SGG	8.2 ± 5.0	54.5 ± 3.3	7.2 ± 3.9	63.6 ± 2.8
SGH	8.3 ± 5.7	51.4 ± 4.6	9.4 ± 5.3	49.1 ± 3.8
SGI	24.5 ± 4.6	69.6 ± 1.2	7.8 ± 5.5	68.9 ± 10.1
SGK	6.1 ± 2.7	40.4 ± 6.3	NA	9.9 ± 3.7
SGO	38.8 ± 4.9	89.6 ± 1.5	46.8 ± 8.2	73.8 ± 8.5
SGBu	49.9 ± 3.3	95.7 ± 0.8	42.7 ± 4.3	86.5 ± 1.8
OST-car	94.4 ± 0.9	100	87.2 ± 4.3	98.9 ± 1.8

SGBu, *n*-butanol extract from *Siparuna glycycarpa*.
OST-car, OST-carboxylate.
NA, not applicable. Influenza not titrated, because cell monolayer was damaged due to viral growth.

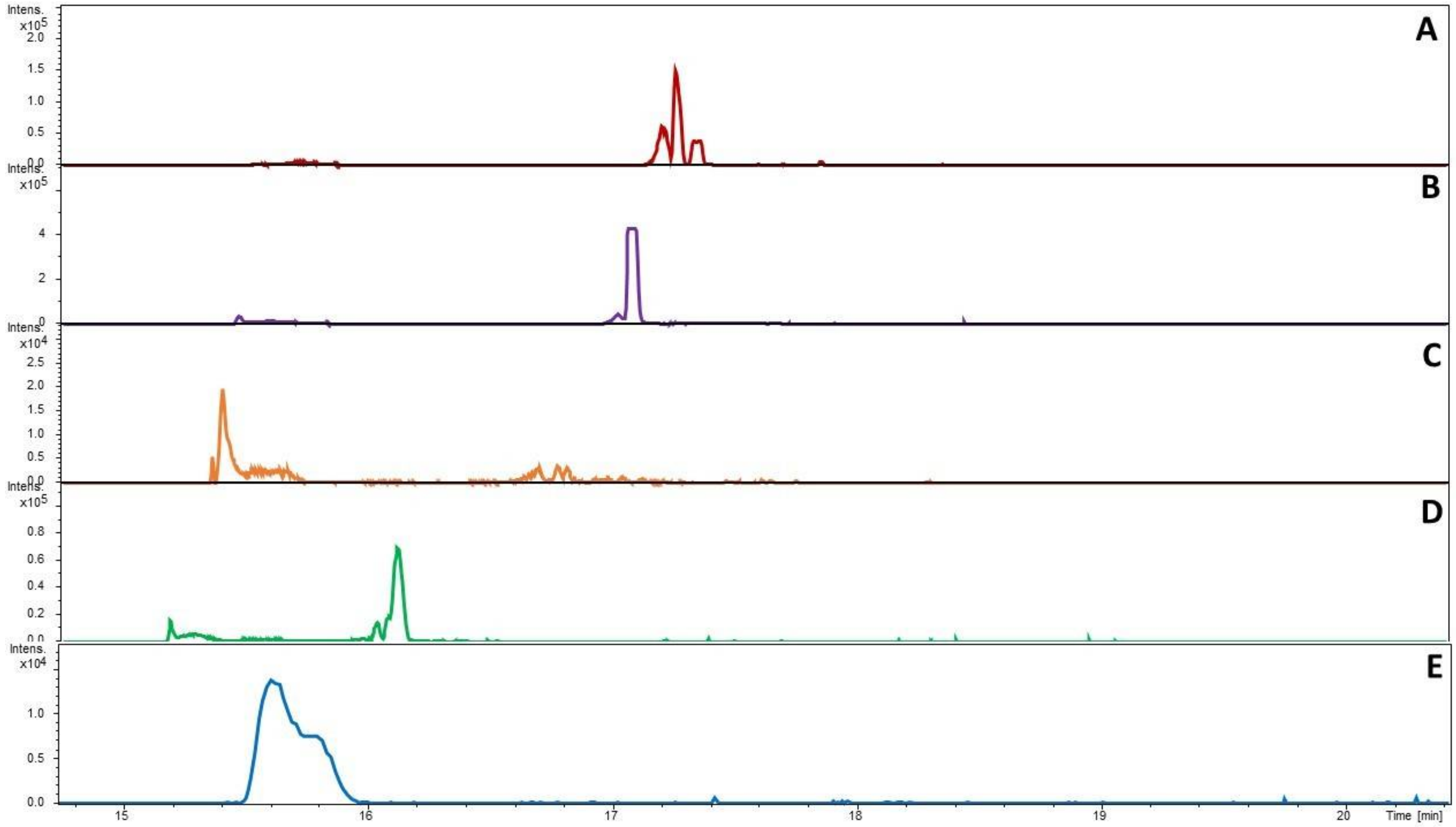

Supplementary Figure S6. (A) SGO, (B) SGD, (C) SGA and (D) SGC LC-HRMS/MS analysis in positive ionization mode. (E) SGO LC-HRMS/MS analysis in negative ionization mode.

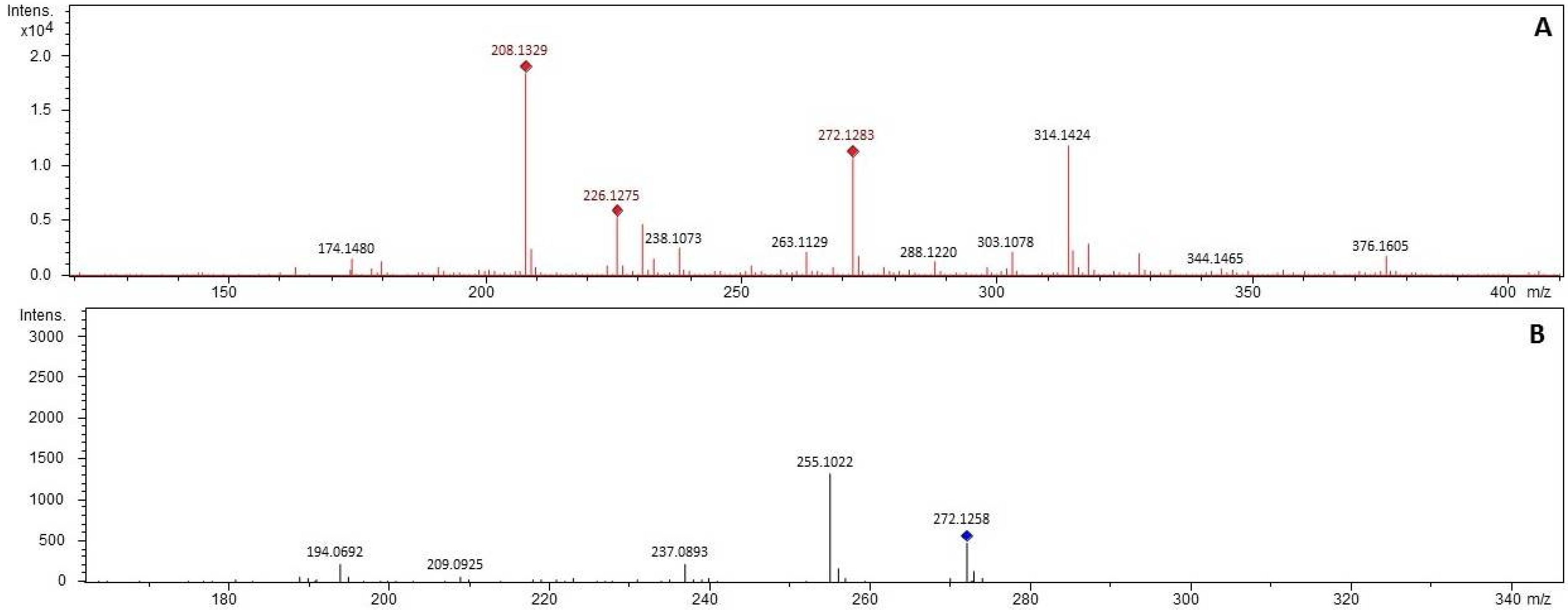

Supplementary Figure S7. (**A**) MS spectrum with *m/z* 272.1283 $[M+H]^+$ and (**B**) MS^2 spectrum of demethyl-coclaurine **1**.

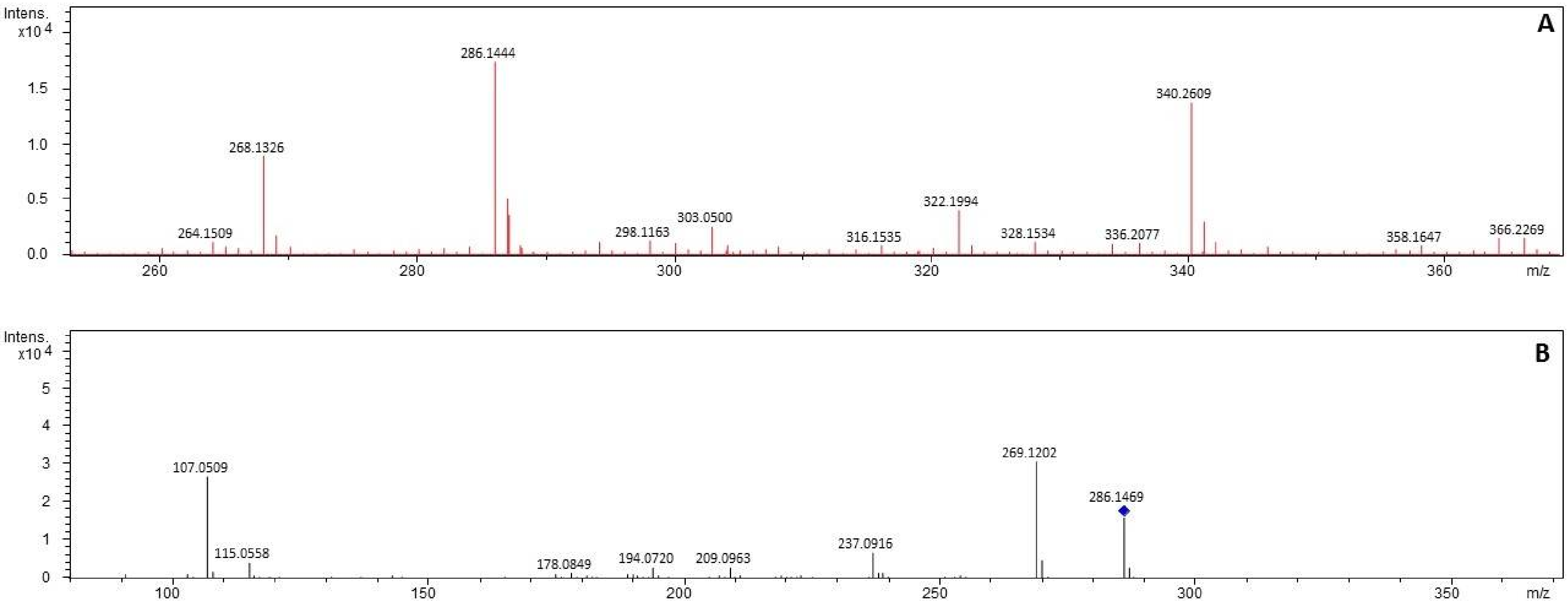

Supplementary Figure S8. (**A**) MS spectrum with *m/z* 286.1444 $[M+H]^+$ and (**B**) MS^2 spectrum of coclaurine **2**.

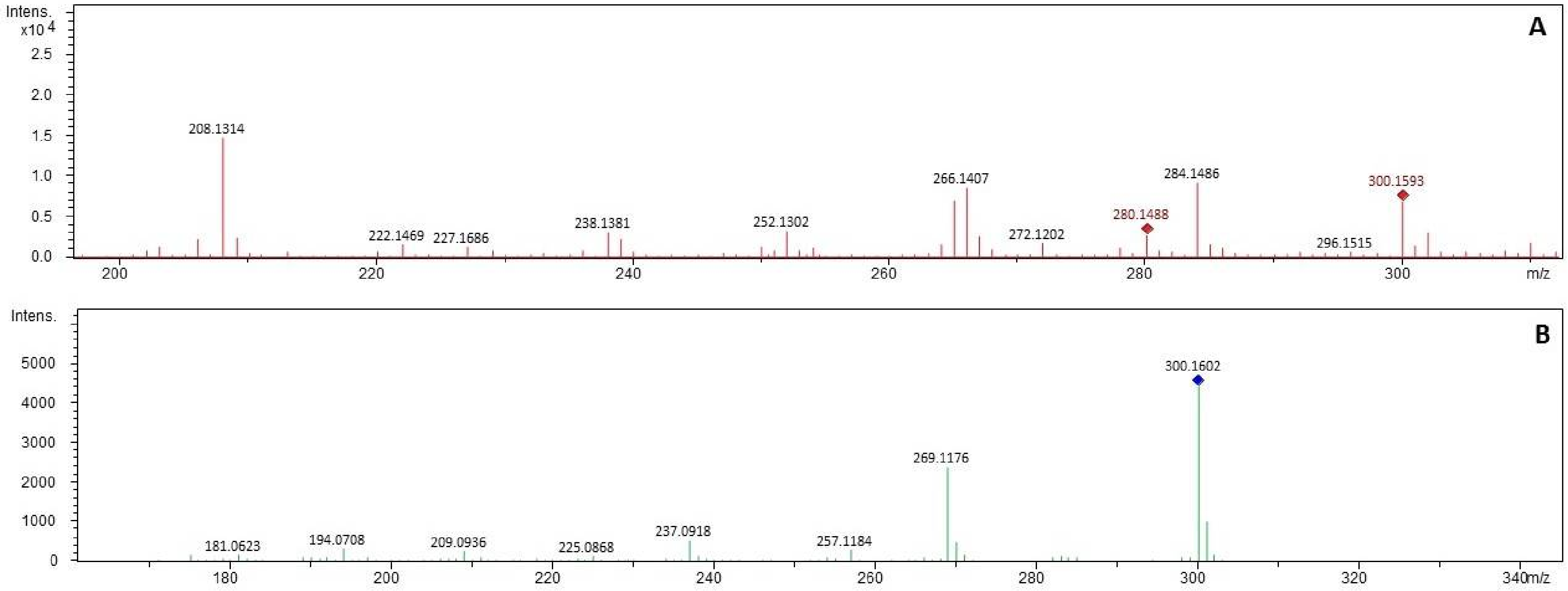

Supplementary Figure S9. (**A**) MS spectrum with *m/z* 300.1593 $[M+H]^+$ and (**B**) MS^2 spectrum of *N*-methylcoclaurine **3**.

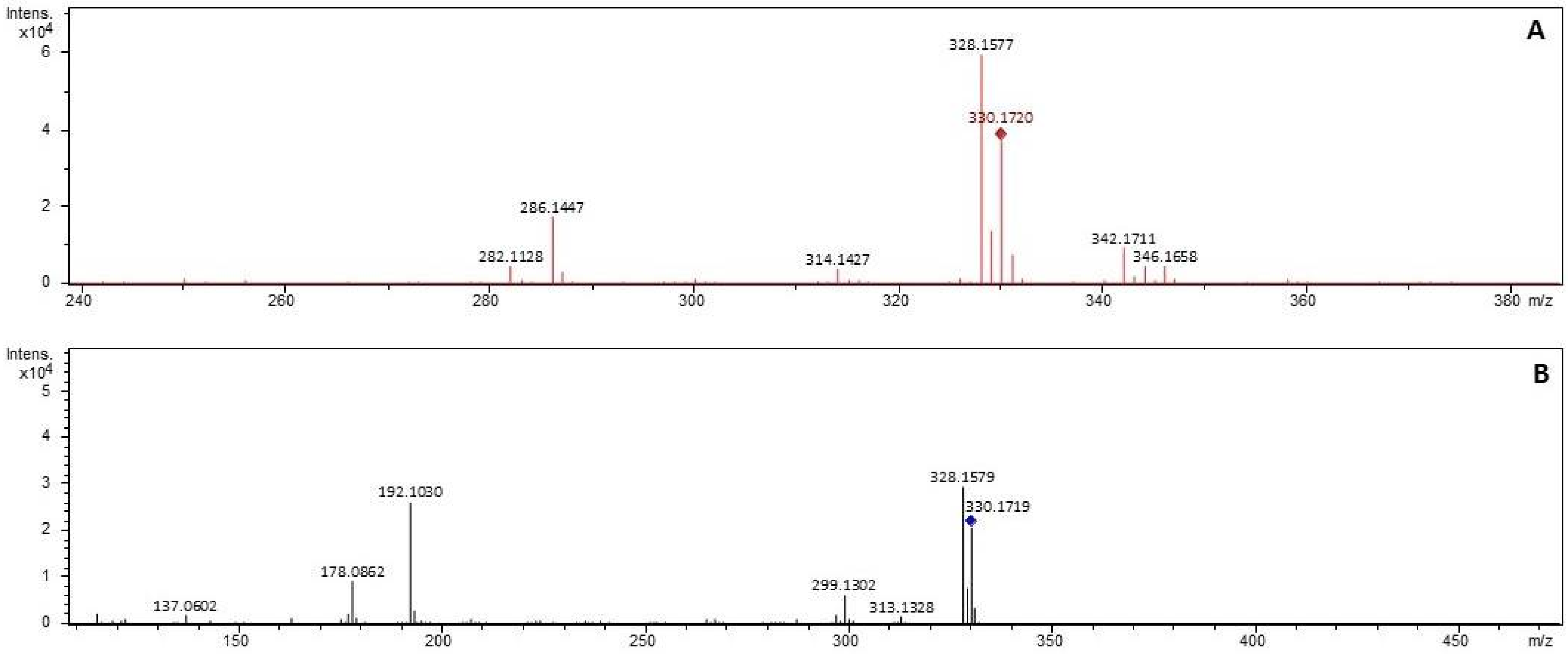

Supplementary Figure S10. (**A**) MS spectrum with *m/z* 330.1720 $[M+H]^+$ and (**B**) MS^2 spectrum of reticuline **4**.

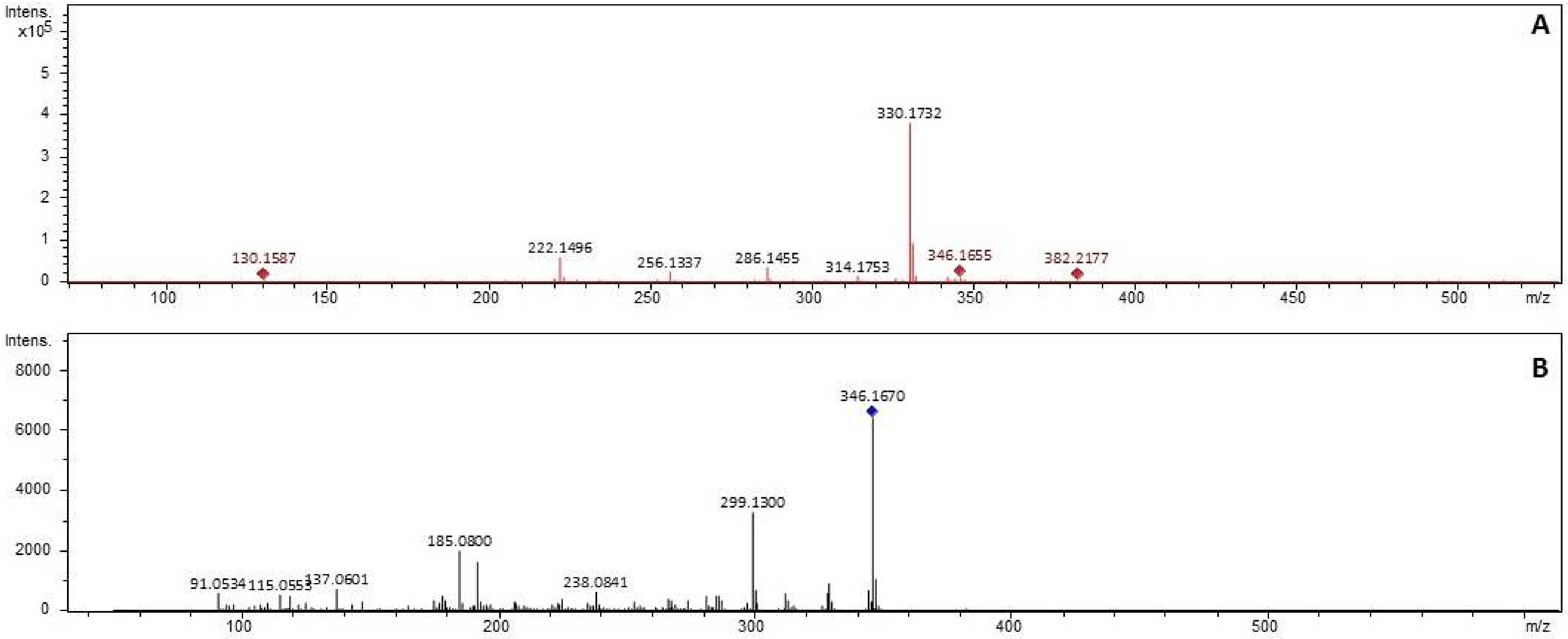

Supplementary Figure S11. (**A**) MS spectrum with *m/z* 346.1655 $[M+H]^+$ and (**B**) MS^2 spectrum of reticuline *N*-oxide **5**.

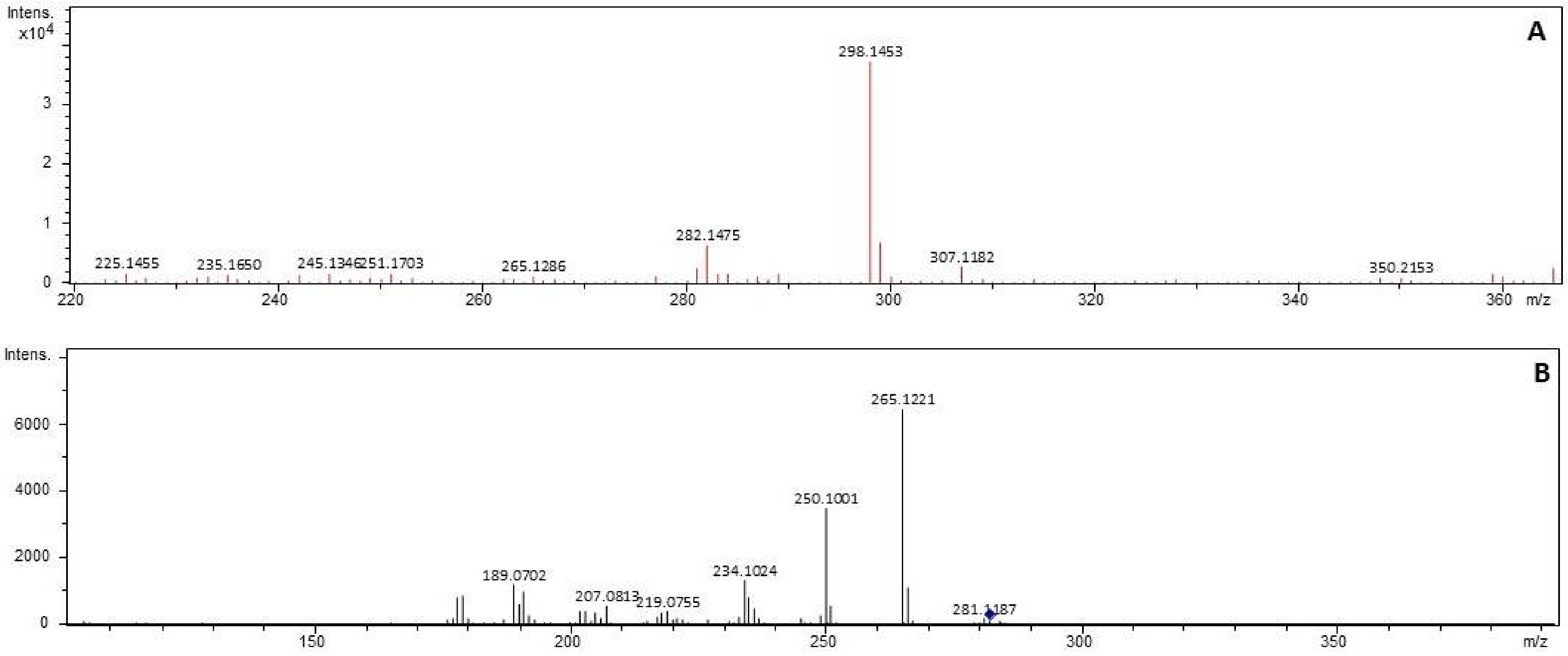

Supplementary Figure S12. (**A**) MS spectrum with *m/z* 282.1475 $[M+H]^+$ and (**B**) MS^2 spectrum of nornuciferine **6**.

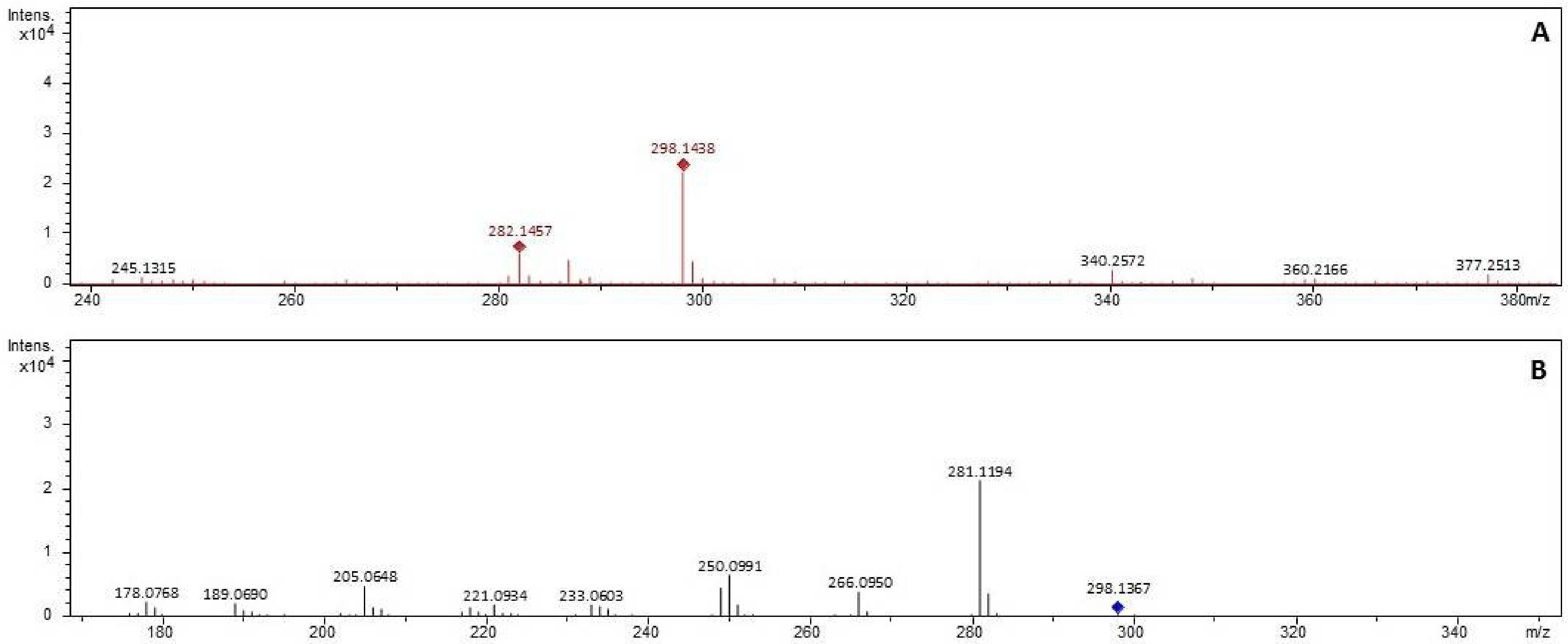

Supplementary Figure S13. (**A**) MS spectrum with *m/z* 298.1438 $[M+H]^+$ and (**B**) MS^2 spectrum of isopiline **7**.

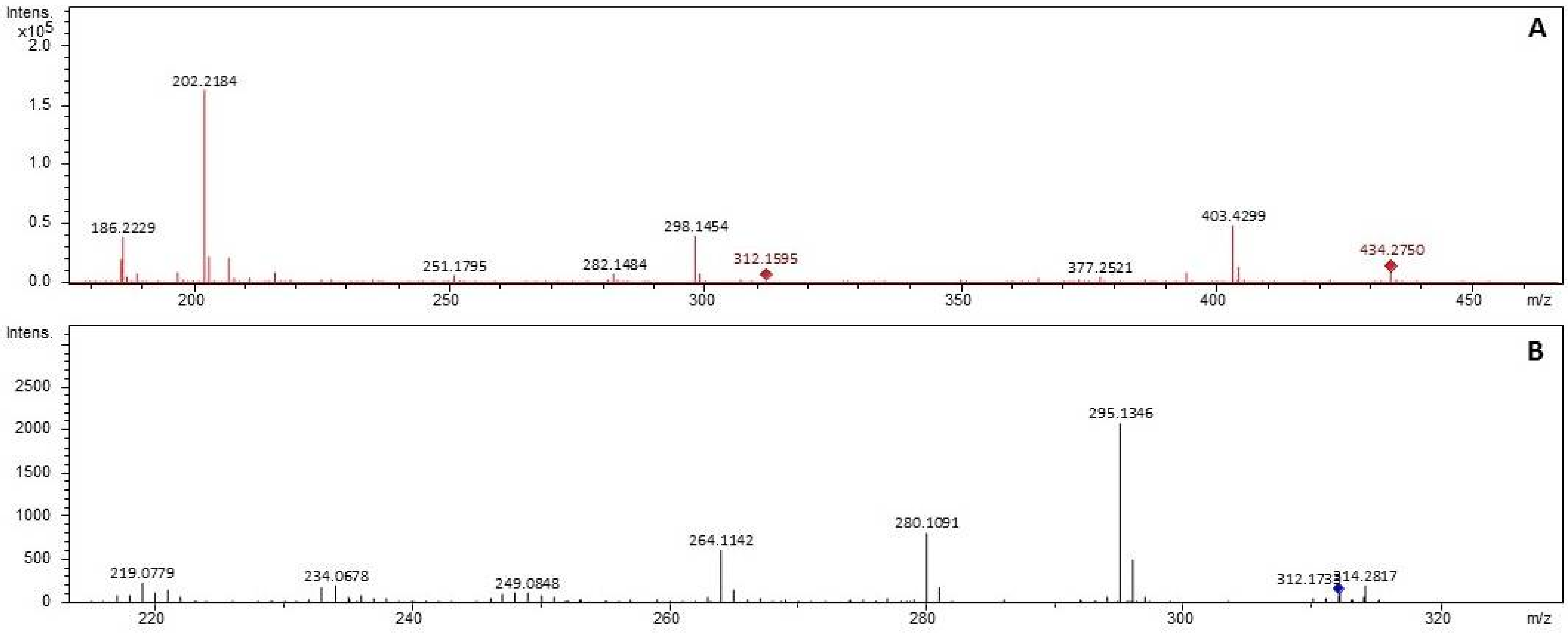

Supplementary Figure S14. (**A**) MS spectrum with *m/z* 312.1595 $[M+H]^+$ and (**B**) MS^2 spectrum of *O*-methylisopiline **8**.

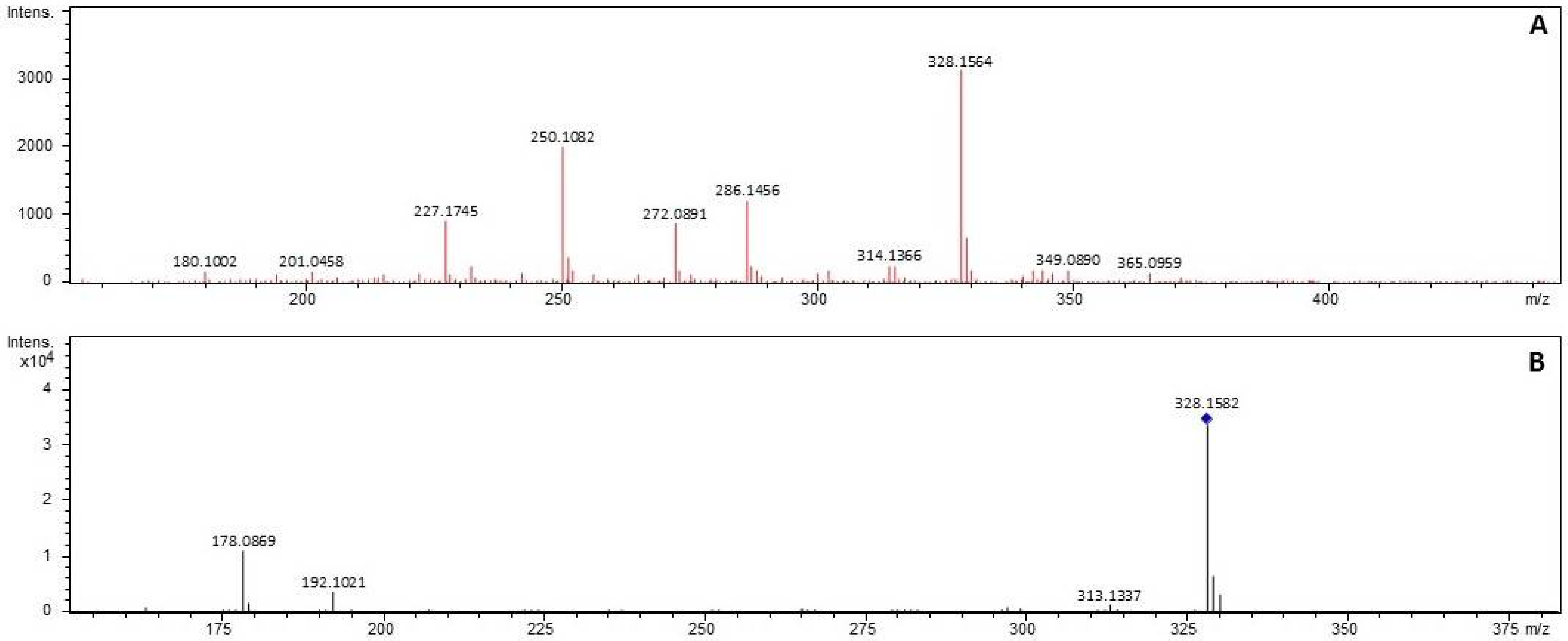

Supplementary Figure S15. (**A**) MS spectrum with *m/z* 328.1564 $[M+H]^+$ and (**B**) MS^2 spectrum of stepholidine **9**.

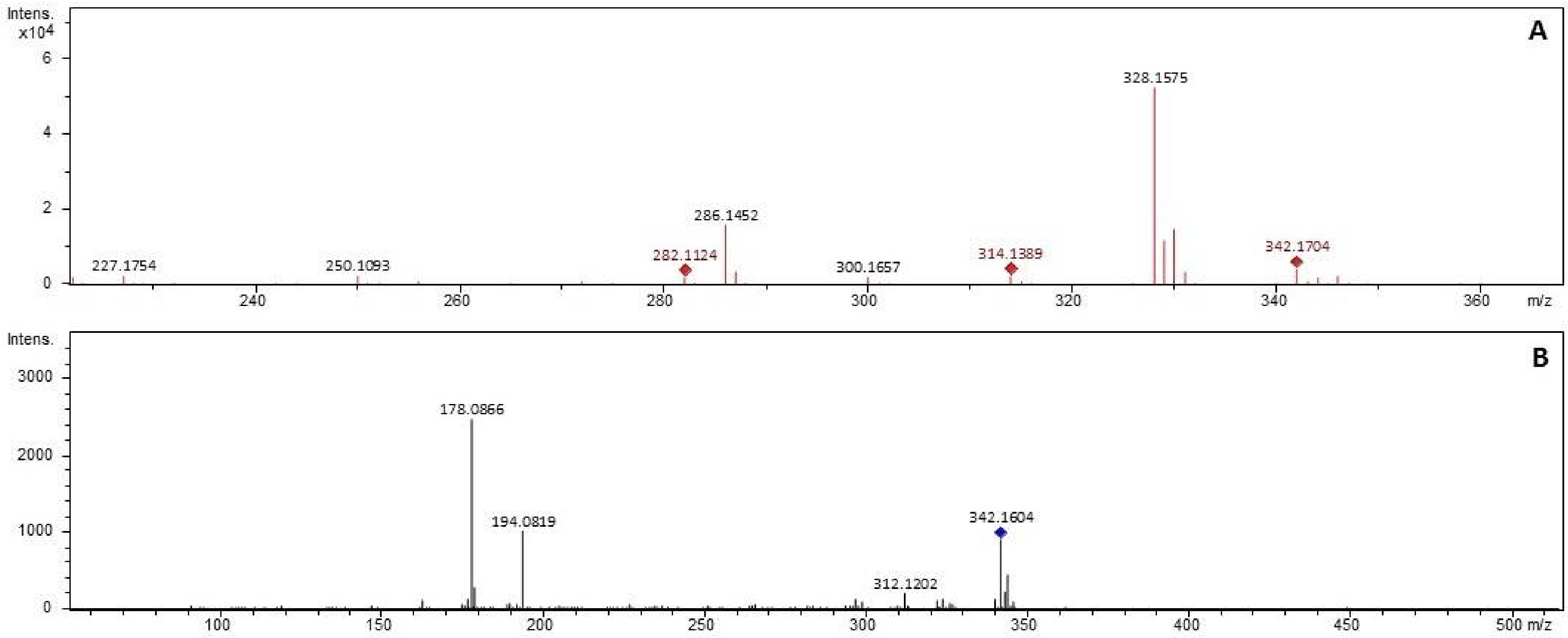

Supplementary Figure S16. (**A**) MS spectrum with *m/z* 342.1704 $[M+H]^+$ and (**B**) MS^2 spectrum of isocorypalmine **10**.

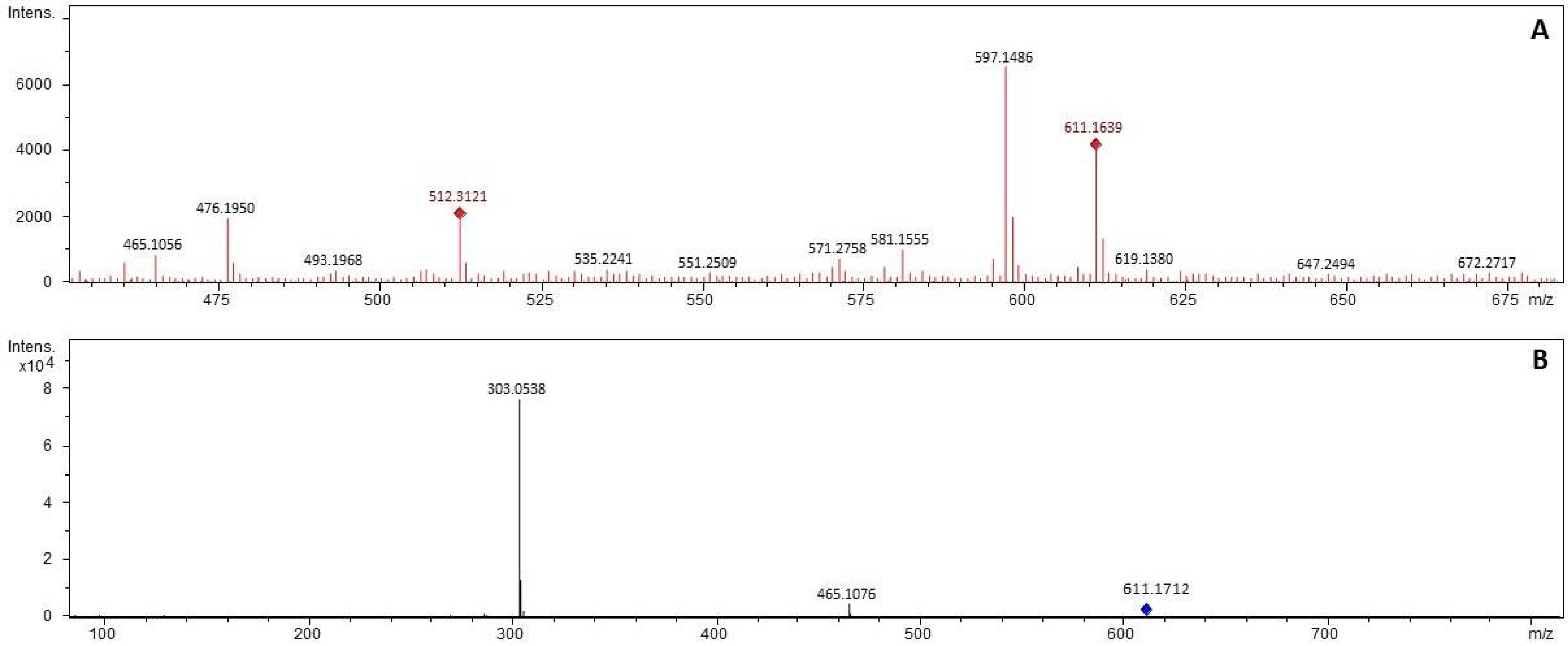

Supplementary Figure S17. (**A**) MS spectrum with *m/z* 611.1639 $[M+H]^+$ and (**B**) MS^2 spectrum of quercetin-3-*O*-rhamnoside-7-*O*-glucoside or quercetin-3-*O*-glucoside-7-*O*-rhamnoside **11**.

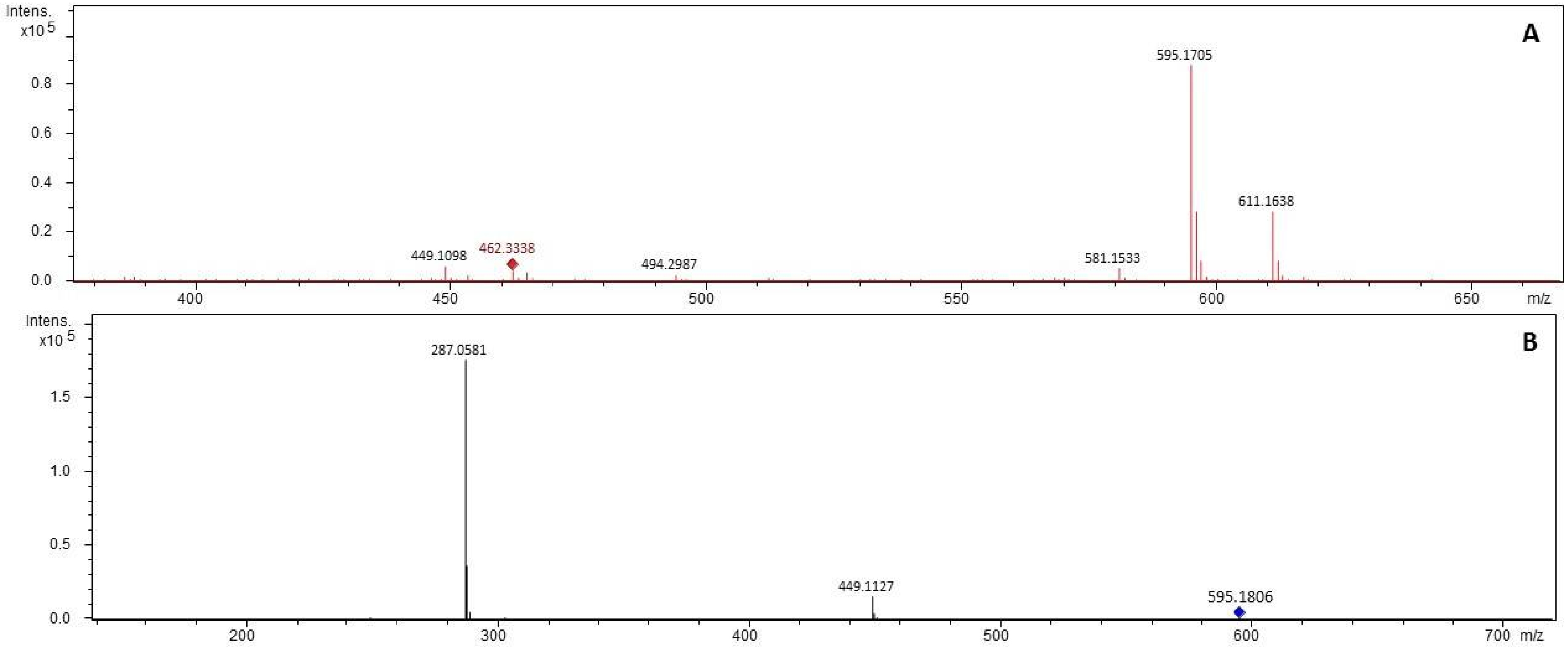

Supplementary Figure S18. (**A**) MS spectrum with *m/z* 595.1705 $[M+H]^+$ and (**B**) MS^2 spectrum of kaempferol-3-*O*-glucoside-7-*O*-rhamnoside **12**.

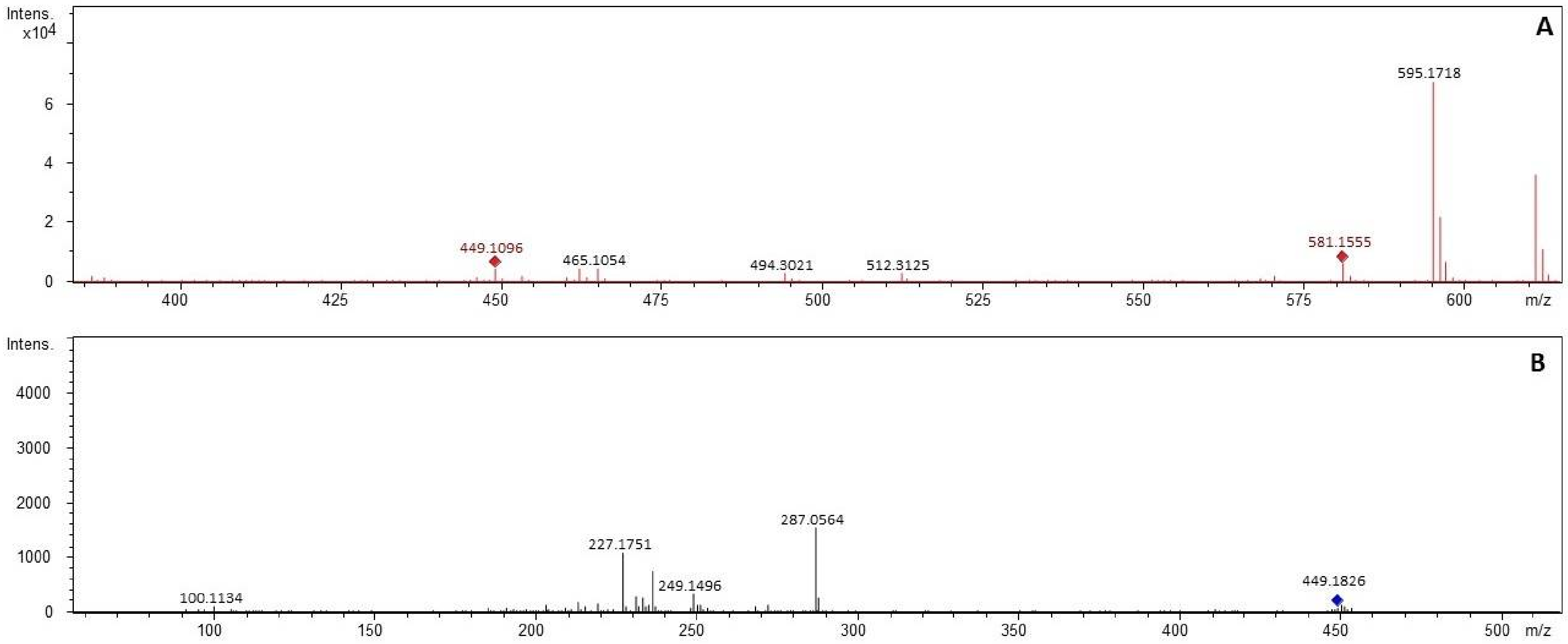

Supplementary Figure S19. (**A**) MS spectrum with *m/z* 449.1096 $[M+H]^+$ and (**B**) MS^2 spectrum of kaempferol 3-*O*-glucoside **13**.

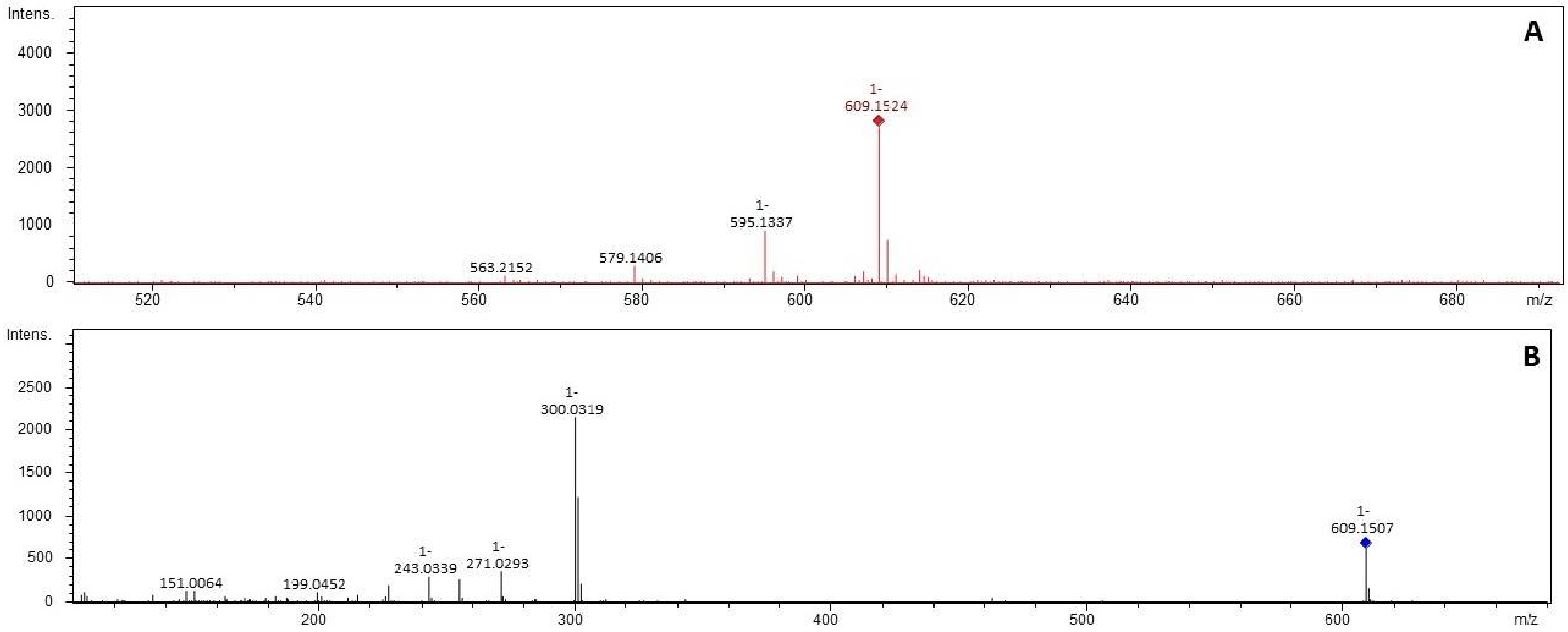

Supplementary Figure S20. (**A**) MS spectrum with *m/z* 609.1524 $[M\text{-}H]^-$ and (**B**) MS^2 spectrum of quercetin-3-*O*-rutinoside (Rutin) **14**.

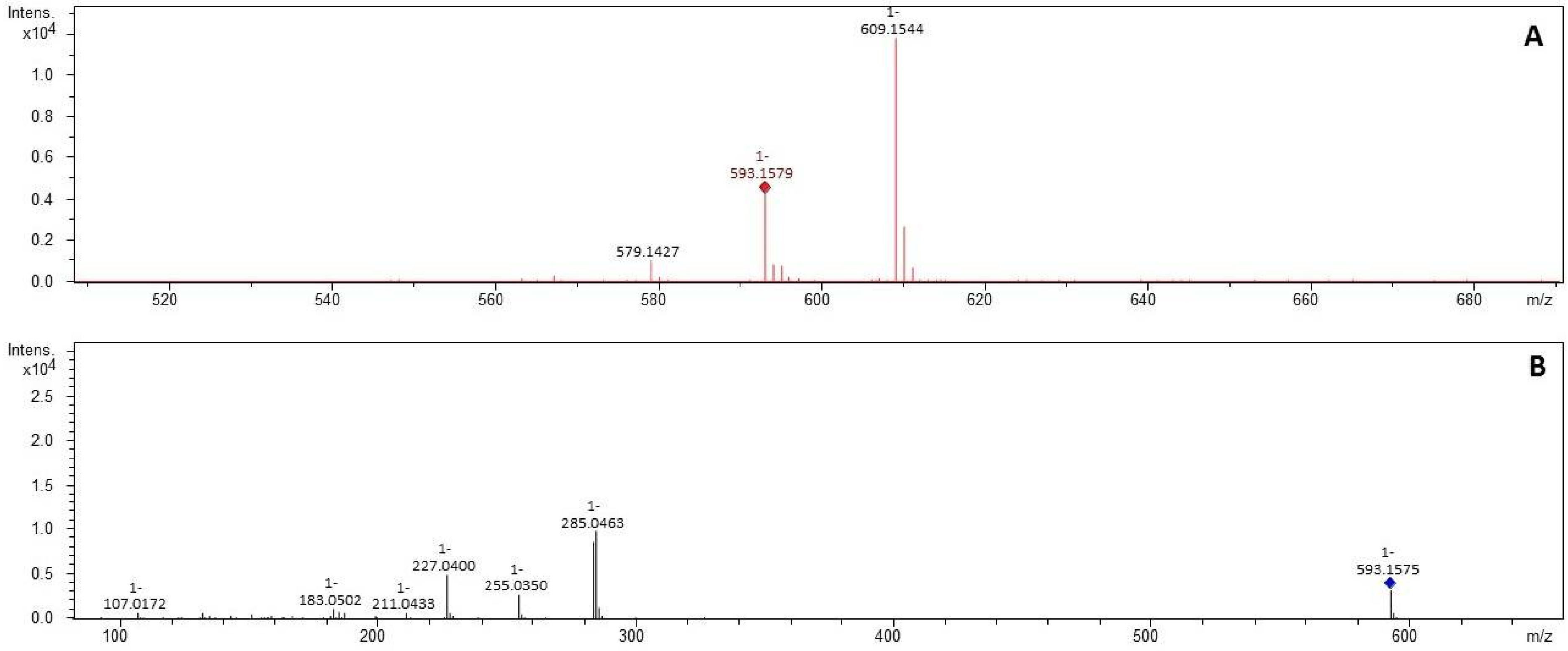

Supplementary Figure S21. (**A**) MS spectrum with *m/z* 593.1579 [M-H]$^-$ and (**B**) MS^2 spectrum of kaempferol-3-*O*-rutinoside **15**.

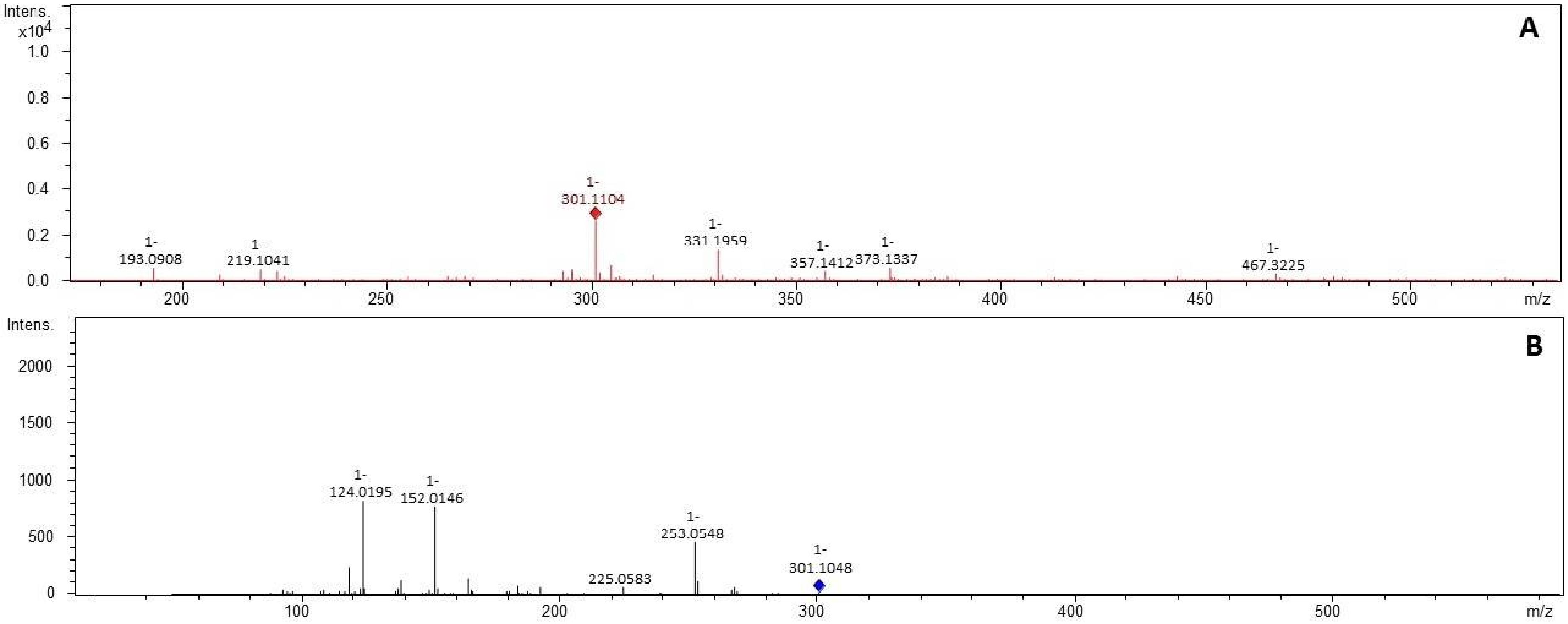

Supplementary Figure S22. (**A**) MS spectrum with *m/z* 301.1104 $[M-H]^-$ and (**B**) MS^2 spectrum of 2’,6’-dihydroxy-4,4’-dimethoxydihydrochalcone **16**.

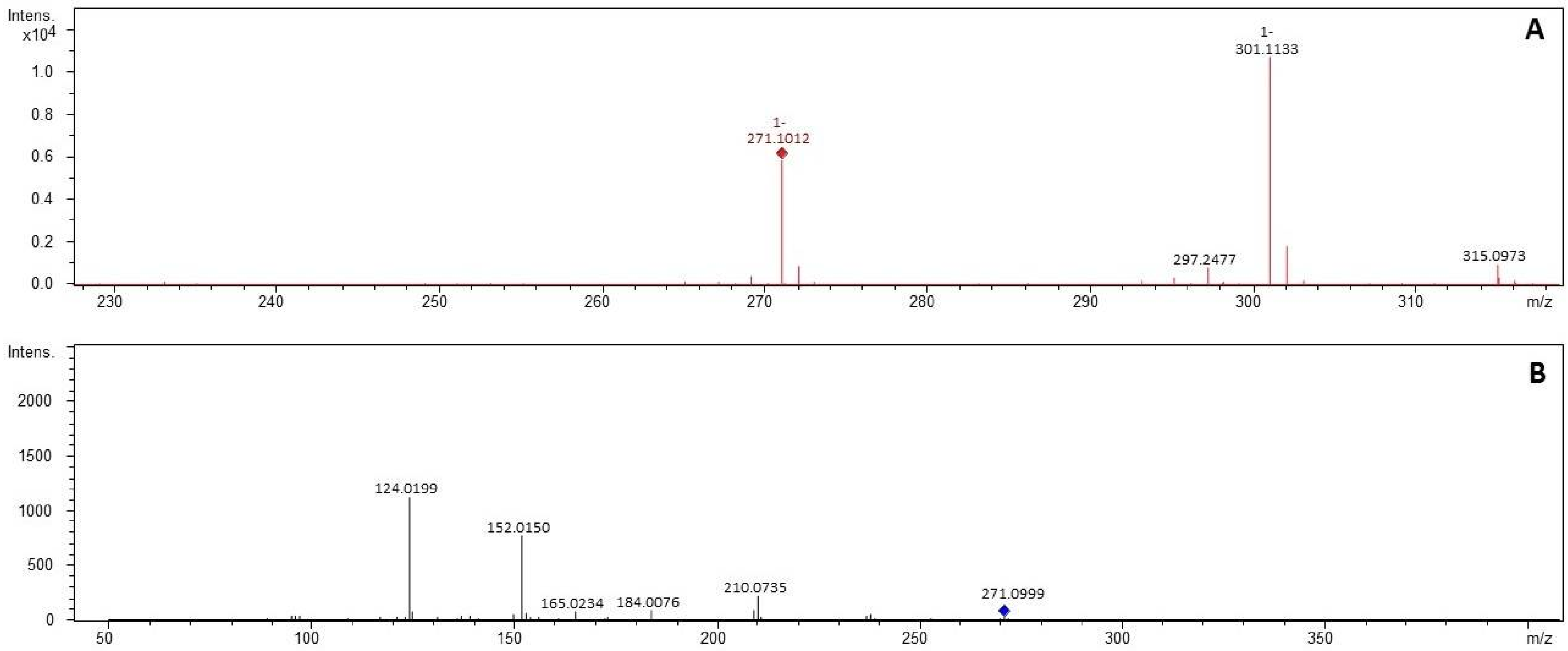

Supplementary Figure S23. (**A**) MS spectrum with *m/z* 271.1012 [M-H]$^-$ and (**B**) MS^2 spectrum of 2',6'-dihydroxy-4'-methoxy-dihydrochalcone **17**.

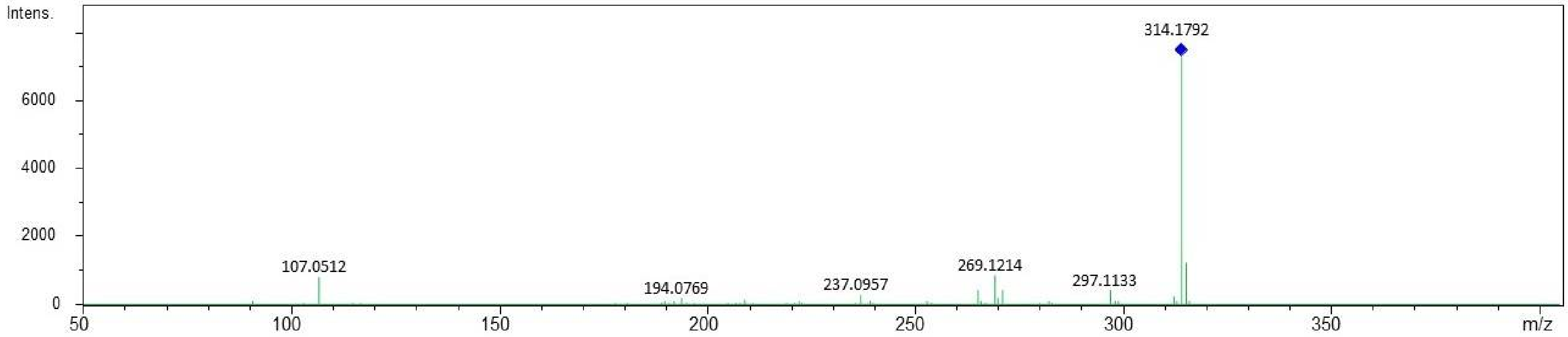

Supplementary Figure S24. MS^2 spectrum of magnocurarine *m/z* 314.1792 [M]$^+$.

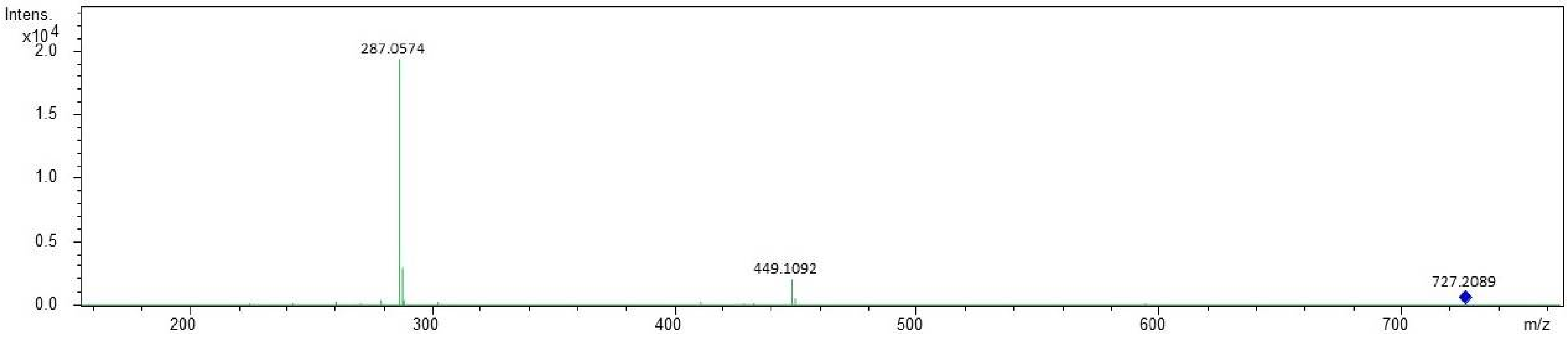

Supplementary Figure S25. MS^2 spectrum of kaempferol-3-*O*-hexose-*O*-deoxyhexose-*O*-pentoside *m/z* 727.2089 [M+H]$^+$.

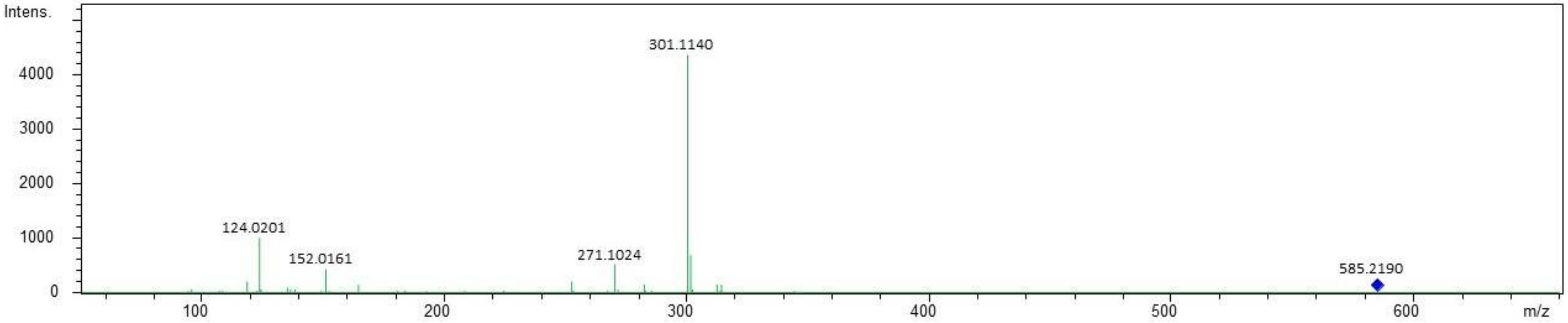

Supplementary Figure S26. MS^2 spectrum of quercetin-3-*O*-(2’’-*O*-galloyl)-pentoside *m/z* 585.2190 $[M-H]^-$.

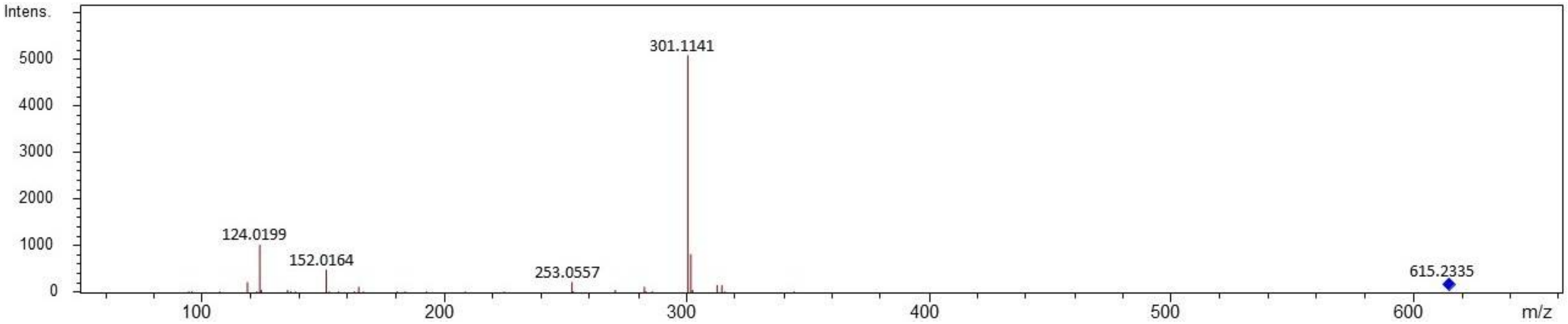

Supplementary Figure S27. MS^2 spectrum of quercetin-3-*O*-(6’’-*O*-galloyl)-β-galactopyranoside *m/z* 615.2335 $[M-H]^-$.
